# Altered Plasma, Urine, and Tissue Profiles of Sulfatides and Sphingomyelins in Patients with Renal Cell Carcinoma

**DOI:** 10.3390/cancers14194622

**Published:** 2022-09-23

**Authors:** Robert Jirásko, Jakub Idkowiak, Denise Wolrab, Aleš Kvasnička, David Friedecký, Krzysztof Polański, Hana Študentová, Vladimír Študent, Bohuslav Melichar, Michal Holčapek

**Affiliations:** 1 Department of Analytical Chemistry, Faculty of Chemical Technology, University of Pardubice, 53210 Pardubice, Czech Republic; 2 Laboratory for Inherited Metabolic Disorders, Department of Clinical Biochemistry, University Hospital Olomouc, Faculty of Medicine and Dentistry, Palacký University, 77900 Olomouc, Czech Republic; 3 Wellcome Sanger Institute, Wellcome Genome Campus, Cambridge CB10 1SA, UK; 4 Department of Oncology, Faculty of Medicine and Dentistry, University Hospital, Palacký University, 77900 Olomouc, Czech Republic; 5 Department of Urology, Faculty of Medicine and Dentistry, University Hospital, Palacký University, 77900 Olomouc, Czech Republic

**Keywords:** cancer biomarkers, sulfatide, sphingomyelins, lipidomics, classification models, early detection, renal cell carcinoma

## Abstract

**Simple Summary:**

Renal cell carcinoma (RCC) is among the most common cancer types in both men and women, and its early detection significantly improves survival. Minimally-invasive blood- or urine-based tests may increase the RCC detection rate, especially before patients develop symptoms. Here, we report significant changes in concentrations of sulfatides and sphingomyelins in plasma and urine in RCC patients compared to healthy controls. For the first time, we present findings that similar alterations appear in the lipid profiles of body fluids and tissues in patients. We observe gradual changes in sulfatide and sphingomyelin concentrations with increasing tumor stage and grade. We built binary classifiers that detect RCC based on plasma and urine lipidome dysregulations, and we show that the plasma lipidome alterations enable distinguishing between early-stage RCC and controls. Our results demonstrate the considerable potential of lipid screening in biofluids for RCC detection and monitoring in clinical settings.

**Abstract:**

Purpose: RCC, the most common type of kidney cancer, is associated with high mortality. A non-invasive diagnostic test remains unavailable due to the lack of RCC-specific biomarkers in body fluids. We have previously described a significantly altered profile of sulfatides in RCC tumor tissues, motivating us to investigate whether these alterations are reflected in collectible body fluids and whether they can enable RCC detection. Methods: We collected and further analyzed 143 plasma, 100 urine, and 154 tissue samples from 155 kidney cancer patients, together with 207 plasma and 70 urine samples from 214 healthy controls. Results: For the first time, we show elevated concentrations of lactosylsulfatides and decreased levels of sulfatides with hydroxylated fatty acyls in body fluids of RCC patients compared to controls. These alterations are emphasized in patients with the advanced tumor stage. Classification models are able to distinguish between controls and patients with RCC. In the case of all plasma samples, the AUC for the testing set was 0.903 (0.844–0.954), while for urine samples it was 0.867 (0.763–0.953). The models are able to efficiently detect patients with early- and late-stage RCC based on plasma samples as well. The test set sensitivities were 80.6% and 90%, and AUC values were 0.899 (0.832–0.952) and 0.981 (0.956–0.998), respectively. Conclusion: Similar trends in body fluids and tissues indicate that RCC influences lipid metabolism, and highlight the potential of the studied lipids for minimally-invasive cancer detection, including patients with early tumor stages, as demonstrated by the predictive ability of the applied classification models.

## 1. Introduction

Malignant neoplasms of the urinary system are associated with high rates of mortality and morbidity, and constitute a major public health problem. RCC represents the most common type of kidney cancer, and its incidence has increased globally in the last decade [1,2,3,4]. The principal subtype of RCC is clear cell (cc) RCC (>75%), with papillary (pap) RCC (15%) and chromophobe (ch) RCC (<5%) being less common.

Early diagnosis improves survival in RCC. Widespread use of abdominal imaging, e.g., ultrasound or computed tomography (CT), has resulted in early detection of RCC in many cases. While a renal mass can be discovered incidentally during abdominal ultrasonography performed for different complaints, no laboratory tests are able to distinguish between RCC and a benign kidney mass. In many cases, RCC is diagnosed late when metastatic disease is present and the prognosis is poor [5]. There are no circulating biomarkers for detecting RCC, such as alpha-fetoprotein in hepatocellular carcinoma, carcinoembryonic antigen and carbohydrate antigen (CA) 19-9 in gastrointestinal malignancies, or CA125 and HE4 in ovarian cancer [6]. Therefore, a non-invasive laboratory test to facilitate RCC diagnosis remains an unmet medical need.

Lipidomic analysis may provide information about ongoing processes in the organism. Reliable lipidomics can serve as support for the detection of cancer [7], the elucidation of biochemical mechanisms associated with tumor growth [8], and the development of new drugs [9]. For example, sphingomyelins, together with ceramides, phospholipids, and glycerolipids, have shown the potential to detect kidney, breast, pancreatic, and prostate cancer [10,11]. Sulfatides, a class of low-abundance sphingolipids, seem to be a good target for such research as well [12]. Sulfatides participate in adhesion to functional proteins, the maintenance and modification of ion channels, and the induction of cell differentiation [13]. The importance of sulfatide metabolism and sulfotransferase gene expression for the morphological differentiation of tumor and metastatic potentials of colorectal, ovarian, or renal carcinomas has been shown in numerous reports [14,15,16,17,18].

In our previous work, we compared 157 tumor and autologous nonmalignant tissues from 80 patients with RCC [19], and reported decreased concentrations of monohexosyl sulfatides (SHexCer) with additional hydroxyl in the ceramide part in the tumor tissue compared to non-neoplastic autologous tissue. These observations are consistent with the results reported by Kim et al. [14]. We found elevated levels of sulfatides composed of two hexosyl units (SHex2Cer) in tumor tissues compared to adjacent nonneoplastic tissues. These results are in agreement with previously described enhanced cerebroside sulfotransferase activity in RCC cells [12,17,20,21]. Recent reports have indicated that lactosylsulfatides are involved in cellular adhesion, and they are suspected to play an important role in the metastasis of hepatocellular carcinoma [22].

Significantly altered sulfatide profiles are usually observed in tumor tissues or cells because of the high local concentrations of studied lipids. However, only a few studies have been published on sulfatide analysis in the cells and tissues of RCC patients [14,19]. To the best of our knowledge, no work on the quantitation of sulfatides in body fluids of RCC patients has been reported to date in the literature.

Here, we compare sulfatide and sphingomyelin profiles of plasma and urine samples taken from RCC patients and controls without cancer. We investigate whether changes in the sulfatide and sphingomyelin profiles of tumor tissues are reflected in body fluids, which may pave the way for developing a low- or non-invasive diagnostic test.

## 2. Materials and Methods

### 2.1. Patients and Sample Collection

Plasma and urine samples of kidney cancer patients and healthy controls were obtained at the Department of Oncology, Faculty of Medicine and Dentistry, Palacký University and University Hospital Olomouc, Czech Republic (Data S1A,B). We obtained 154 tissue samples, including 77 kidney tumor parts and 77 adjacent nonmalignant kidney tissue parts from 77 RCC patients (Data S1C), from the Department of Urology, Palacký University, Faculty of Medicine and Dentistry and University Hospital, Olomouc, Czech Republic [19]. Control samples were obtained from volunteers without cancer records (the only exclusion criterion). For cancer patients, disease was confirmed by biopsy, surgical resection, or histology. All cancer patients and controls were of Caucasian ethnicity. Samples were collected at the same place and processed the same way. The study was approved by the Ethics Committee of the University Hospital Olomouc and the Medical Faculty of Palacký University in accordance with Ethical Principles for Medical Research Involving Human Subjects (Helsinki Declaration). All subjects signed informed consent. Blood and urine collection was performed after overnight fasting with the recommendation to avoid fat-rich meals and alcohol consumption the evening before the collection. Blood and urine from cancer patients were obtained before surgical operation and treatment. No repeated collections were performed for healthy controls or cancer patients. The samples were stored at −80 °C, transported from the hospital to the analytical laboratory on dry ice, and subsequently stored again at −80 °C until sample processing.

### 2.2. Sample Processing

Solvents for extraction were purchased from Sigma-Aldrich (St. Louis, MI, USA). For lipid extraction from 25 μL of plasma, a modified Folch method with a chloroform–methanol–water mixture was employed [11]. The samples were spiked before extraction with a mixture of internal standards obtained from Avanti Polar Lipids (Alabaster, AL, USA) to achieve final concentrations of 0.1 nmol of SHexCer 18:1;O2/12:0 and 43.3 nmol of SM 18:1;O2/12:0 per mL of plasma. For urine samples, reversed-phase solid-phase extraction (SPE) was performed. Briefly, 2 mL of human urine together with 3 μL of the mixture of internal standards dissolved in methanol (SHexCer 18:1;O2/12:0 of concentration 1.7 μg/mL and D4 taurocholic acid of concentration 16.7 μg/mL) were loaded into a 200 mg tC18 cartridge (Sep-Pak Vac, 37–55 µm particle size) (Waters, Milford, MA, USA), resulting in concentrations of 0.04 nmol for SHexCer 18:1;O2/12:0 and 0.55 nmol for D4 taurocholic acid per ml of urine. SPE columns were previously primed with 3 mL of methanol followed by 3 mL of water. The columns were washed with 3 mL of water and the studied lipids were further eluted with 3 mL of methanol. The eluates were collected, evaporated under a gentle stream of nitrogen, and redissolved in the mixture of 600 µL of methanol before measurement. Extraction of lipids from tissue samples was as described in our previous article [19].

### 2.3. Mass Spectrometry Analysis

Mass spectra were measured using an ultrahigh-resolution MALDI LTQ Orbitrap XL mass spectrometer (Thermo Fisher Scientific, Waltham, MA, USA) equipped with a nitrogen UV laser (337 nm, 60 Hz). The LTQ Orbitrap instrument was operated in negative-ion mode in a normal mass range *m/z* 400–2000, and the mass resolution was set to R = 100,000 (full width at half maximum definition, at *m/z* 400) for all full scan mass spectrum experiments. We dissolved 9-AA (Sigma-Aldrich, St. Louis, MO, USA) in a mixture of methanol (Sigma-Aldrich) and water mixture (4:1, *v/v*) to provide a concentration of 5 mg/mL for plasma samples or 10 mg/mL for urine samples, which was then mixed with lipid extracts (1:1, *v/v*). Deionized water was prepared with a Milli-Q Reference Water Purification System (Molsheim, France). The deposited volume of each sample on the target plate was 1 μL; deposition was followed by dried droplet crystallization. Each sample was deposited on a MALDI plate five times and measured in negative polarity mode. Zig-zag sample movement with a step size of 250 μm was used during individual data acquisition. The laser energy corresponded to 2.7 μJ and two microscans/scan, with two laser shots per microscan at 36 different positions accumulated for one measurement to achieve a reproducible signal.

### 2.4. Data Processing

Each measurement was represented by five average MALDI-MS spectra (five repetitions) with thousands of *m/z* values for each sample. Subsequently, automatic peak assignment was performed and *m/z* peaks were matched with deprotonated molecules ([M-H]^−^ for sulfatides and sterol sulfates (StS) or [M-CH_3_]^−^ for SM) from a database created during the identification procedure using the Excel macro script available on figshare (https://figshare.com/s/d01d43e862b7e03adbfe, (accessed on 21 December 2021)). This peak assignment resulted in the generation of a list of the present *m/z* of the studied lipids along with the median intensities (raw intensities) for each measured sample (Data S1A–C). For all three types of samples, we further calculated both relative (Data S1D–F) and molar (Data S1G–I) concentrations. In the case of urine and tissue samples, after normalization of native lipid species’ intensities to the internal standard, large analytical variance of the obtained concentrations was observed within the studied samples. Therefore, we decided to further investigate relative concentrations through direct comparison of all three types of matrices (plasma, urine, and tissue). Only lipids which met two inclusion criteria were used to calculate relative concentrations. The first criterion (inclusion criterion 1) was that the evaluated lipid species must be present at least in 50% of samples of individual data sets (plasma, urine, and tissue) in both groups (i.e., controls and cancer cases). The second criterion (inclusion criterion 2) was that the coefficient of variation of the raw intensities (Data S1A–C) of the lipids in the measured quality control (QC) samples must be less than 35%. In the case of urine samples, we excluded 27 selected subjects (24 patients and 3 controls) because more than 50% of the lipids were below the limit of quantitation (LOQ) or the IS signal was below the LOQ due to the high matrix effect (inclusion criterion 3). For relative quantitation, zero values (see Data S1A–C) were replaced by the value corresponding to the lowest reproducible intensity (8000) for all lipids. The signal intensities of each lipid (feature) were related to the sum of all intensities for all lipid species within a particular class, i.e., SM, sulfatides, and StS, and multiplied by 100 for each sample separately to calculate the relative percentage (i.e., the percent abundance of lipid species within individual lipid classes).

### 2.5. Statistical Data Analysis

All calculations and visualizations were carried out using Microsoft Excel (ver. 2111), R free software environment (ver. 4.1.2), SIMCA statistical software (ver. 13.0.0.0), and CorelDRAW Graphics Suite (ver. 23.5.0.506). In the first step, the distribution of variables in the dataset was usually observed based on the density plots generated using the ggplot2 library. Then, medians and interquartile ranges (IQR) were calculated for all the variables within groups using the rstatix library. Fold change (FDR) was computed via the robust Hodges–Lehmann type fold change estimator. To obtain this, we computed all possible pairwise ratios between the feature values in two groups, and then the median of those ratios was returned. To compare differences between two independent groups, we used the Mann–Whitney U test for plasma and urine, while its equivalent for paired samples was applied in the case of tissue samples (rstatix package). The comparison of controls and cases split into stages or grades (multiple group analyses) was performed using the Kruskal–Wallis test (rstatix library). When the Kruskal–Wallis test indicated differences, the Conover post hoc test was applied to determine which pairs of groups differed significantly (PMCMRplus library). The rank–biserial correlation coefficient effect size was computed for the Mann–Whitney two-sample test using the rcompanion library and the epsilon squared effect size was computed for the Kruskal–Wallis test using the ggstatsplot library. For the presence/absence analysis, first, contingency tables were prepared in which lipids with an intensity >8000 were classified as “1” (present) and otherwise as “0” (absent). Fisher’s exact test was used to analyze contingency tables concerning lipids detected in plasma and urine samples. In the case of tissues, both parts were taken from the same subject, and we considered the following pairs: lipid present (or absent) in nontumor/tumor tissue at once, i.e., (1, 1) or (0, 0); lipid present only in nontumor tissue (1, 0); or lipid present only in tumor tissue (0, 1). McNemar’s test was applied in the next step (exact 2 × 2 library) in order to analyze the contingency tables for tissues. Spearman correlations were calculated to investigate the relationships between age and lipid concentrations and BMI and lipid concentrations (psych library). Volcano plots were prepared with the use of the EnhancedVolcano library. Differences between groups were visualized in the form of box plots for skewed distributions (litteR and ggplot2 libraries). The construction of such box plots is presented and explained in Hubert and Vandervieren [23]. All *p*-values were corrected for multiple comparison using Benjamini–Hochberg assessment of the False Discovery Rate (FDR). In the next step, the data were log-transformed and Pareto scaled (tidymodels, tidySpectR libraries). Principal Component Analysis was performed using SIMCA statistical software. For circular dendrograms, the Euclidean distances were calculated first, then Ward’s clustering algorithm was applied (phangorn library). Dendrograms were generated by applying the ggtree and ggplot2 libraries. The z-scores for each transformed concentration were calculated before plotting the heat map. Logistic regression with ridge penalty (glmnet library) was applied to classify plasma and urine samples. First, the classification of RCC cases (T) and controls (N) was performed for both types of samples. Using plasma samples, we built models for the classification of controls (N) and patients with early-stage RCC (T1-2) as well as for controls (N) and patients with late-stage RCC (T3-4). In the case of plasma samples, 33 lipid concentrations were included in the model along with BMI, and gender, while for urine samples the model included concentrations of 33 lipids, BMI, and gender. Missing BMI values were substituted by the median within each group. Then, data were split into training (60%) and testing set (40%), log-transformed, and Pareto scaled. For plasma samples, upsampling was used prior to model training in order to handle data imbalances (caret library). A five-fold cross-validation approach was applied to prepare the model (caret library). The model with the value of the lambda regularization parameter corresponding to the highest area under the receiver operating characteristic curve (AUC) was selected for each classification (caret library). Receiver operating characteristic curves (ROC) were generated (pROC library) in order to assess the performance of each model and the corresponding AUC values were calculated. The confusion matrices were computed using the caret library; 95% confidence intervals were computed for AUC and accuracy using the caret library and for specificity and sensitivity using the bdpv library. ROC curves were prepared for sample classification based on the individual lipids only. The AUCs from both this analysis and from fold changes were subsequently used to generate the network visualization of alterations in lipid profiles of RCC vs. controls in the Cytoscape software, with the color saturation reflecting fold changes and the size of each circle the AUC for each lipid. All statistical calculations supporting our data are listed in Supplementary Data S2.

## 3. Results

### 3.1. Study Design and Analytical Validation

**Figure 1:**
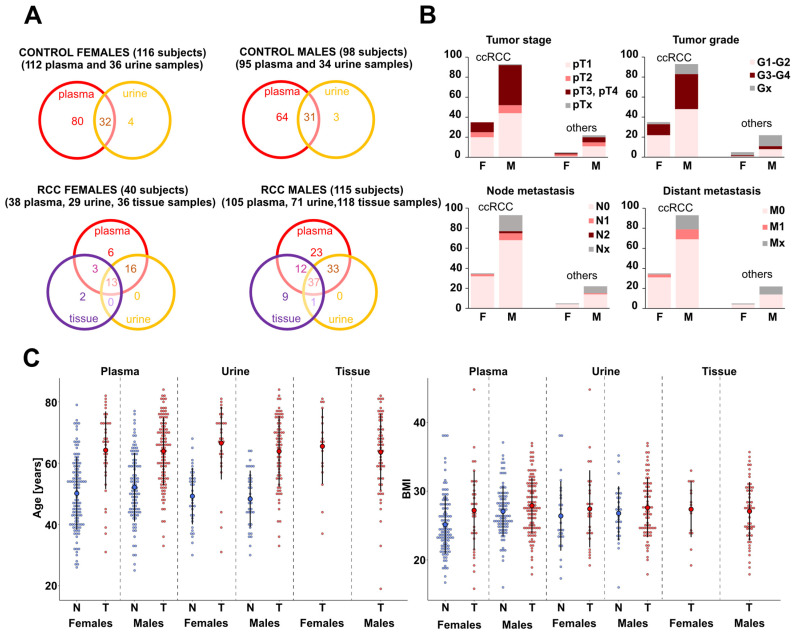
Overview of number of samples and clinical information. (**A**) Venn diagrams showing the number of individual plasma, urine, and tissue samples obtained from control volunteers and kidney cancer patients (separately illustrated for females and males). To obtain the number of tissue samples, it is necessary to multiply the numbers in the purple circles by two, as both tumor and nontumor types of tissue were obtained from each patient. (**B**) Stacked bar charts reflecting the TNM tumor classification [24] of cancer patients. The graphs show the number of patients grouped by individual tumor stage, tumor grade, and type of node and distant metastases, separately shown for males (M) and females (F) (see Supplementary Data S1 for more details). The first two bar charts represent ccRCC patients and the other two represent patients with other kidney cancer types. (**C**) Dot plots illustrating the age and BMI distribution of control volunteers (N, blue dots) and cancer patients (T, red dots) in the case of plasma, urine, and tissue samples.

A significant part of the cancer samples were ccRCC (*n* = 128) taken from patients with different tumor stages (Figure 1B), including patients with metastatic disease. Patients with other RCC subtypes were studied as well, including fifteen cases of papRCC, four cases of chRCC, three cases of multilocular cystic renal cell carcinoma (mcRCC), and five cases of patients with other kidney tumors (Figure 1B and Data S1).

In total, we collected and further investigated 143 plasma, 100 urine, and 154 tissue samples (tumor and non-tumor adjacent tissue parts from 77 patients) from 155 kidney cancer patients together with 207 plasma and 70 urine samples from 214 control volunteers (subsequently referred to as controls). An overview of sample types from cancer patients and controls is illustrated in Figure 1A, and information about collected types of samples for individual subjects (cancer cases and controls) is listed in Supplementary Data S1A–G in the column titled ‘Measured types of samples*‘*. Samples of two types of body fluids (plasma and urine) were investigated for 99 patients with RCC, among whom tissue samples were evaluated in 50 cases. The higher number of male cancer patient samples in this study reflects the fact that RCC patients are more often male and typically have larger tumors with higher stage and grade than females.

The analytical methodology we used was validated in order to ensure high data quality (Supplementary Data S3). The validation results show that the methodology can be used to quantify sulfatides, sphingomyelins, and sterol sulfates. QC samples were included in the sequence measurements to control instrument performance and data acquisition quality (Figure S1 and Supplementary Data S3). We established inclusion criteria for measured lipids before constructing statistical models or calculating relative concentrations (see Section 2.4). Data distribution was checked for all quantified lipids within the studied groups, resulting in the application of nonparametric tests.

We follow the lipid nomenclature in the LIPID MAPS system [25] and the shorthand notation for lipid structures [26,27]. A detailed explanation is listed in Supplementary Data S3.

### 3.2. Alterations in the Plasma Lipid Profiles of Cancer Patients

**Figure 2:**
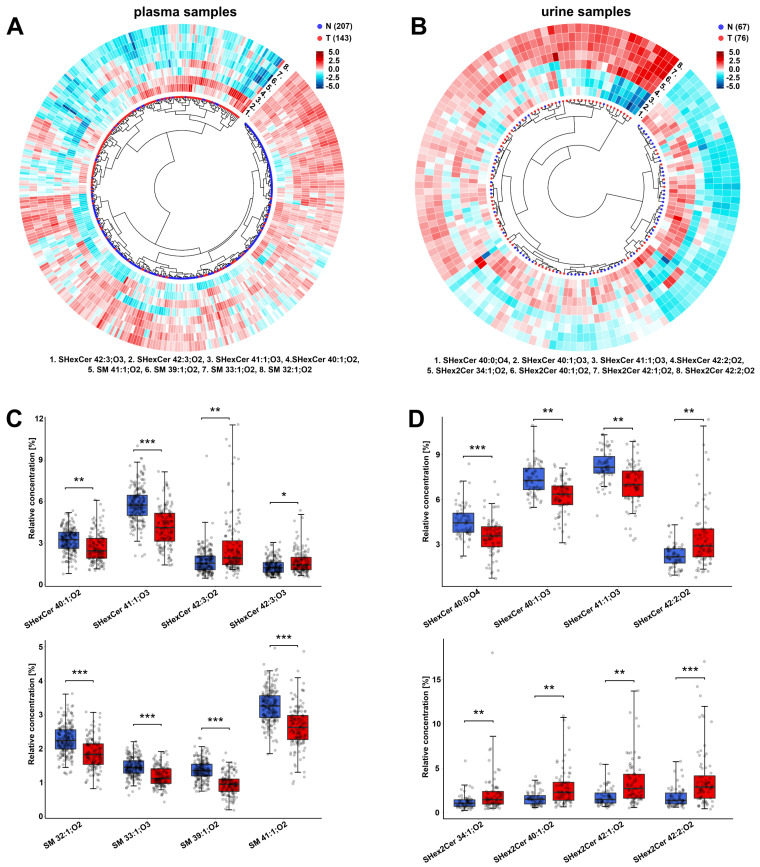
Important dysregulations observed in studied plasma and urine samples. Circular dendrograms with heat map calculated based on the relative concentrations of (**A**) the four most significant SM species and four most significant SHexCer species in 350 plasma samples or (**B**) the four most significant SHex2Cer and four most significant SHexCer species in 143 urine samples, demonstrating the classification of controls (N) and RCC cancer cases (T). Data were log-transformed and Pareto scaled, then the Euclidean distances were calculated and Ward’s clustering method was selected. The heatmap was generated using z-score scaling. Box plots of the relative concentrations of these significant lipids for controls and patients with RCC in the case of (**C**) plasma samples and (**D**) urine samples. The importance marked above the box plots includes the fold change (robust Hodges–Lehmann type fold change estimator), effect size, and FDR from the Mann–Whitney U test: *** large, ** medium, * small significance (see detailed explanation in Supplementary Data S2A,D).

As a first step, we compared the relative concentrations of 33 lipids (Data S1D) measured in 143 plasma samples obtained from kidney cancer patients and 207 plasma samples collected from controls. Statistical analysis results for all 33 lipids are listed in Supplementary Data S2A. We selected the four most statistically significant lipids for both studied lipid classes (SHexCer and SM) based on the fold change, effect size, and false discovery rate. These lipids were used to generate a circular dendrogram (Figure 2A), showing good classification of controls and cancer cases. In general, most of the studied lipids are downregulated in cancer patients compared to controls, as demonstrated by the volcano plot (Figure S1B) and selected box plots (Figure 2C). The decreased concentrations of SM 41:1;O2, SM 40:1;O2, SM 39:1;O2, SM 38:1;O2, SM 33:1;O2 and SM 32:1;O2 are the most significant changes among sphingomyelins.

Sulfatides containing one double bond, most of which feature additional hydroxyl groups (abbreviated as SHexCer XX:1;O3), such as SHexCer 40:1;O3, SHexCer 41:1;O3, and SHexCer 42:1;O3 together with SHexCer 40:1;O2, show significant downregulation in cancer patients. In turn, sulfatides with more double bonds, such as SHexCer 42:3;O2, SHexCer 42:3;O3, and SHexCer 42:2;O2, are upregulated in cancer patients.

Sulfatides with two hexosyl units (lactosylsulfatides) were usually below LOQ in plasma, except for SHex2Cer 42:2;O2, however, higher concentrations than LOQ were observed for only 26 samples. In the case of lipids which were detected in fewer than 50% of RCC cases or controls, we decided to use the present/absent approach (Supplementary Data S2C); such lipids were not used for the calculation of relative concentrations, as described earlier (inclusion criterion 1). In this way, we investigated whether lipids occurred more frequently in the plasma of controls or cancer patients (N or T). The results of this analysis are presented using radar charts (Figure S2A). We observed that very low abundant sphingomyelins and sulfatides with one double bond and additional hydroxyl groups are generally more commonly present in the plasma of controls than in cancer patients. In turn, SHex2Cer 42:2;O2 is present more often in the plasma of RCC patients (24 cancer patients versus only two controls). In previous research, we have found elevated SHex2Cer 42:2;O2 in tumor tissue [19].

Finally, we examined whether differences in the plasma lipidome could be observed between patients with four subtypes of RCC, namely, ccRCC, papRCC, chRCC, and mcRCC. Only minor or no differences were found, and similar dysregulations were observed for each cancer subtype in the profiles of sphingomyelins and sulfatides (Figure S3). A thorough investigation of the differences between RCC subtypes should be performed due to the limited number of samples collected during this study; however, such research is beyond the scope of the current article.

### 3.3. Alterations in the Urine Lipid Profiles of Cancer Patients

After data filtration (inclusion criterion 3; see Section 2.4), 76 samples taken from RCC patients and another 67 from controls were used to perform a statistical comparison of urine samples (Supplementary Data S2D). To ensure that our exclusion of 27 samples did not significantly affect the reported findings, we compared the statistical results before and after data filtration. No significant differences between the two datasets were observed; the predictive ability is even better when all subjects are used in the classification models, as illustrated in Figure S4.

We quantified eighteen SHexCer and four SHex2Cer in urine. No SM were detected, as the concentrations were below the limit of detection, while eleven sterol sulfates (StS) were quantified (Supplementary Data S1E). A circular dendrogram with a heat map was prepared for eight selected sulfatides (Figure 2B), showing differences between the RCC and control samples. In the urine of patients with RCC, concentrations of lactosylsulfatides are elevated compared to control samples. These differences are presented in the heatmap surrounding the dendrogram (Figure 2B), volcano plot (Figure S1D), and in the form of box plots (Figure 2D). Higher levels of SHex2Cer 34:1;O2, SHex2Cer 40:1;O2, SHex2Cer 42:1;O2, and SHex2Cer 42:2;O2 are the most significant upregulations. Sulfatides with an additional hydroxyl group in the ceramide backbone (SHexCer XX:XX;O3 or SHexCer XX:XX;O4) show the opposite dysregulation, similar to the plasma samples.

The presence/absence analysis results show that hydroxylated sulfatides occur more often in the urine of controls than RCC cases (Figure S2B, Supplementary Data S2E). Another difference between the urine samples of RCC patients and controls is observed in the case of sterol sulfates. In patients with RCC, we observe upregulation of StS 3 and StS 4 and downregulation of StS 8 and StS 11 (Figure S5).

### 3.4. Different Sulfatide and Sphingomyelin Levels in Cancer Patients by Advanced Tumor Stage and Grade

To answer whether the lipid changes observed in both biological fluids are associated with tumor stage and grade, we divided the plasma and urine sample sets into controls, patients with early (T1-T2) tumors, and more advanced tumors (T3-T4). Similarly, we divided samples by grade. The results of our statistical tests are presented in the Supplementary Data S2F,G for plasma samples and S2H,I for urine samples, respectively.

**Figure 3:**
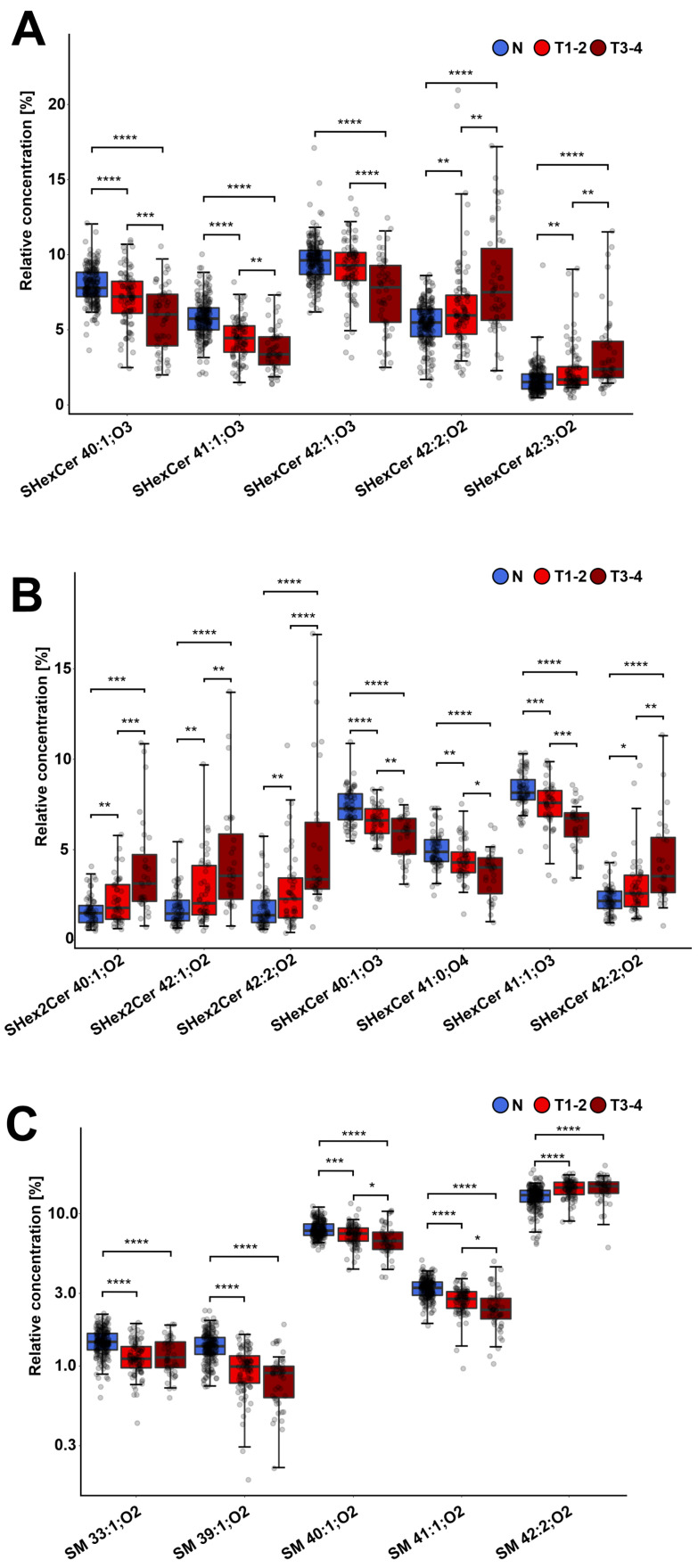
Increasing dysregulation trend of sulfatides and SM for more advanced tumor stages. Box plots of the most statistically significant lipids for controls, patients with tumor stages T1-2, and patients with T3-4: (**A**) sulfatides in plasma samples, (**B**) sulfatides in urine samples, and (**C**) SM in plasma samples. The importance marked above the box plots indicates the results of the Conover post hoc test (FDR): 0.05–0.01 *, 0.01–0.001 **, 0.001–0.0001 ***, 0.0001 and lower ****.

The box plots in Figure 3A,B illustrate a gradual drop in concentrations of the hydroxylated sulfatides SHexCer XX:XX;O3 and SHexCer XX:XX;O4 in plasma and urine samples, respectively, for advanced tumor stage. In contrast, a gradual increase in concentrations of the lactosylsulfatide SHex2Cer XX:XX;O2 and monohexosylated sulfatides SHexCer 42:2;O2 and SHexCer 42:3;O2 was observed with increasing cancer stage. Similarly, lower levels of SM with longer fatty acyls, i.e., SM 39:1;O2, SM 40:1;O2 and SM 41:1;O2, were found in patients with more advanced tumor stages (Figure 3C). These findings show that plasma and urine lipid profiles in RCC differ slightly between patients depending on how advanced the tumor is.

### 3.5. Observed Sulfatide and Sphingomyelin Dysregulations in RCC Patients Are Similar in Body Fluids and Tumor Tissues

To support the hypothesis that the dysregulations observed in plasma and urine reflect cancer progression, we further investigated differences between the lipid profiles of 77 tumor and 77 autologous nonneoplastic tissues of 77 patients (Supplementary Data S1F). The results of statistical tests for 44 quantified lipids, including 20 SHexCer, 12 SHex2Cer, and 12 SM species, are listed in the Supplementary Data S2J.

**Figure 4:**
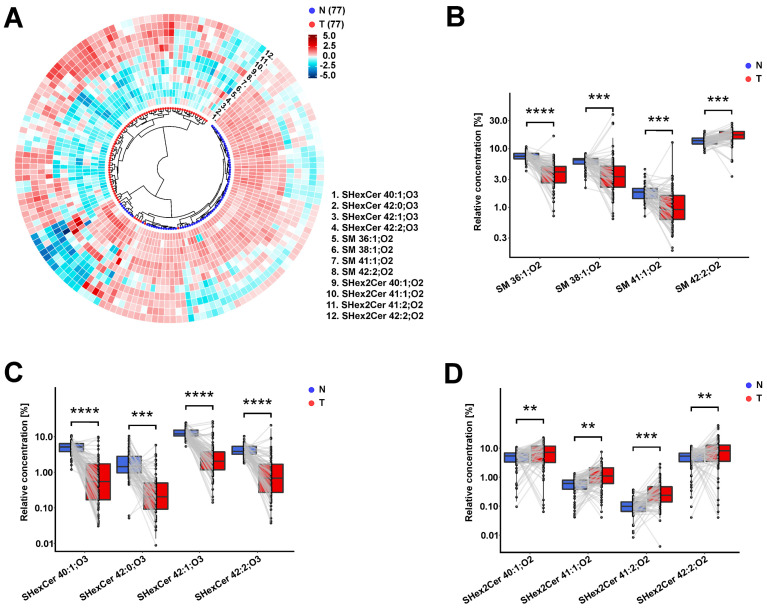
Important dysregulations observed in studied tissue samples: (**A**) Circular dendrogram with heat map calculated based on the concentrations of the four most significant SM, four most significant SHexCer, and four most significant SHex2Cer species, demonstrating classification of tumor (red) and nonneoplastic (blue) tissue samples of 77 RCC patients. Data were log-transformed and Pareto scaled, then the Euclidean distances were calculated and Ward’s clustering method was selected. The heatmap was generated using z-score scaling. (**B**–**D**) Paired box plots of concentrations of four most significant SHexCer, four most significant SHex2Cer, and four most significant SM in tumor tissue and nonneoplastic tissue samples of RCC patients. The importance marked above box plots includes fold change, effect size, and FDR from the Wilcoxon signed-rank test: **** very large, *** large, ** medium (see detailed explanation in Supplementary Data S2J).

When we compared the lipid profiles of the two tissue types, we found similar changes to those observed in body fluids. In the tumor tissues, hydroxylated sulfatides and SM with one double bond, such as SM 41:1;O2, are downregulated. In contrast, the elevated SHex2Cer species SHexCer 42:2;O2, SHexCer 42:3;O2, and SM 42:2;O2 are observed in tumor tissues, as illustrated in the volcano plot in Figure S1F and the paired box plots in Figure 4B–D. These alterations in SHex2Cer, SHexCer, and SM are presented in the circular dendrogram’s heat map (Figure 4A) as well. In the dendrogram, it can be seen that nonneoplastic and tumor tissue samples are well-separated.

The results from the presence/absence analysis for lipids which did not fulfill inclusion criterion 1 in the case of tissue samples are summarized in Supplementary Data S2K. As with the plasma and urine results, we found that hydroxylated sulfatides and SM are present more often in nonneoplastic parts and absent in the tumor tissue.

**Figure 5:**
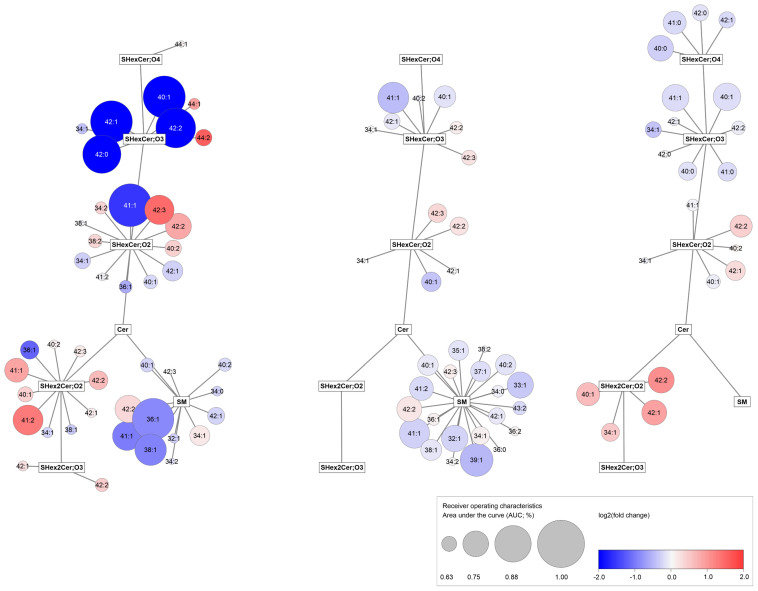
Network visualization of dysregulated lipids in RCC across all three types of samples. The graphs show simplified metabolism of sphingolipids for tissue (**left**), plasma (**middle**), and urine (**right**) samples; Cytoscape software (http://www.cytoscape.org, accessed on 17 December 2021) was used to generate the networks. Circle sizes express the AUC values for binary classifiers constructed for individual lipids (Supplementary Data S2S), while red/blue color saturation represents fold change (T/N).

Then, to see the whole picture, we compared all alterations in lipid profiles found in RCC samples to controls across all three studied materials, as illustrated in network visualization (Figure 5). Similar dysregulation trends in all RCC samples might indicate that lipid alterations in tumor tissue are reflected in the studied body fluids. However, no gradual drop with increasing tumor stage (Supplementary Data S2L,M) or grade (Supplementary Data S2N,O) is observed in the case of tissue samples, as presented in Figure S6. Patients with advanced tumors are at increased risk of developing metastases, and advanced disease might result in stronger differences between their lipid profiles and controls. We compared lipid concentrations in body fluids (plasma and urine) of patients with cancer stage T3 both with and without metastases (Supplementary Data S2P,Q; Figure S7A–C).

### 3.6. RCC Detection Based on Alterations in Plasma and Urine Sphingolipid Profiles

To check the potential of the studied lipids for clinical cancer detection, we divided plasma and urine samples into training and testing cohorts; about 40% of all samples were allocated to the testing set.

For both types of samples, the relative concentrations of 33 lipids were used along with gender and BMI to build the classification models. We selected logistic regression with a ridge penalty. The influence of the clinical parameters on lipid concentrations, including gender, age, and BMI, was analyzed before the model was built. All subjects were split according to gender and then into controls, RCC patients with tumor stage T1-2, and RCC patients with tumor stage T3-4. After splitting the data according to gender, we observed that the respective medians in the box plots shifted slightly, suggesting that gender separation might improve the differentiation of cases from controls (Figure S7D,E). Spearman correlations were calculated between lipid concentrations and age and between lipid concentrations and BMI (Supplementary Data S2R). Most of the obtained correlations indicate relatively weak associations between age and lipid concentrations and between BMI and lipid concentrations, with most being not statistically significant. Moderate positive correlations between BMI and SM 36:0;O2, SM 36:1;O2, and SM 36:2;O2 levels and moderate negative correlations between BMI and SHexCer 42:1;O2, SHexCer 42:2;O2, and SHexCer 42:3;O2 levels may indicate alterations in the metabolism of C18 and C24-based sphingolipids after obesity. In addition, stronger correlations between lipids and BMI for females with T3-4 cancer stages may be associated with the low number of samples available for calculations. Finally, while gender and BMI were included in the model, we removed age because it was not well-balanced between controls and RCC patients (Figure 1).

**Figure 6:**
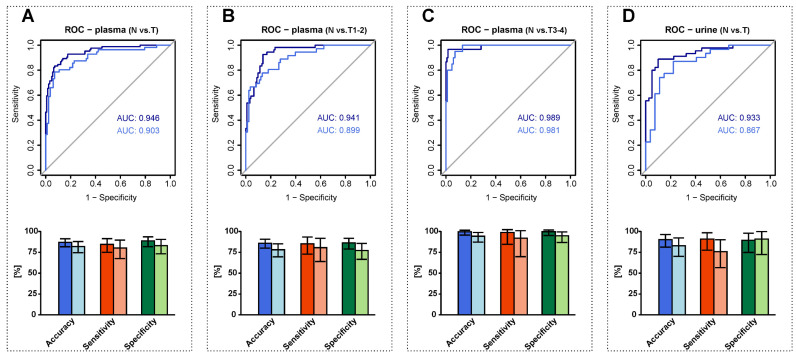
Potential of the studied lipids in case of clinical cancer detection. Receiver operating characteristic (ROC) curves illustrate the diagnostic ability of logistic regression with ridge penalty. The dark blue color of the lines refers to the training data set prediction and the light blue to the testing data set prediction. For each case, the areas under the ROC curves (AUC) are presented in dark blue for the training data set prediction and light blue for the testing data set prediction. The models were constructed for all plasma samples, prediction of RCC (**A**); for plasma samples, prediction of early-stage RCC (**B**); for plasma samples, prediction of late-stage RCC (**C**); and for all urine samples, prediction of RCC (**D**). Bar charts with 95% confidence intervals (CI) represent training accuracy (dark blue), sensitivity (dark red), specificity (dark green), and testing accuracy (light blue), sensitivity (light red), and specificity (light green).

In the first step, we investigated whether alterations in sphingolipid profiles enabled distinguishing between all RCC cases (T) and healthy controls (N). In the case of plasma, the value of the area under the receiver operating characteristic curve (AUC) was 0.946 (95% CI 0.913–0.972) for the training set prediction and 0.903 (95% CI 0.844–0.954) for the testing set prediction. In the case of urine, the AUC was 0.933 (95% CI 0.879–0.980) for the training set prediction and 0.867 (95% CI 0.763–0.953) for the testing set prediction. In the next step, relying on the set of plasma samples, we tested how effectively the constructed models were able to distinguish between controls and individuals with early-stage RCC as well as between controls and individuals with late-stage RCC. The AUC of the model for early-stage RCC prediction was 0.941 (95% CI 0.905–0.970) for training set prediction and 0.899 (95% CI 0.832–0.952) for the testing set prediction. The best performance was with the model prepared for classifying patients with late-stage RCC and healthy controls; the obtained AUC for the training set was 0.989 (95% CI 0.966–1.000) and 0.981 (95% CI 0.956–0.998) for the testing set. The results are visualized in Figure 6, and individual accuracies, sensitivities, and specificities are listed in Supplementary Data S2T.

## 4. Discussion

This work demonstrates that the plasma and urine alterations in sulfatides and sphingomyelins enable distinguishing between RCC cases and controls without cancer based on minimally-invasive blood collection or non-invasive urine collection. The logistic regression models for classifying healthy controls and RCC patients are characterized by good test set sensitivities and specificities, namely, 80.4% and 83.1% for the plasma samples and 74.3% and 88.9% for urine samples, respectively. The diagnosis of malignancy in patients with early-stage cancer is usually challenging, especially in asymptomatic or mildly symptomatic patients. However, early-stage kidney cancer may already cause alterations in lipid metabolism which can be detected based on simple blood or urine lipidome screening. Sulfatide and sphingomyelin profiling in plasma/urine could represent a complementary test to ultrasound examination. Therefore, in the next step, we built models for distinguishing between early-stage RCC and controls based on sulfatide and sphingomyelin profiling in plasma samples. We found that logistic regression with a ridge penalty allowed for the correct classification of 80.6% of cases with early-stage RCC and 77.1% of controls. Although alterations in plasma sphingolipid profiles are less emphasized in early-stage than in late-stage RCC, our results indicate the suitability of plasma sphingolipid profiling for effective differentiation of patients with RCC from controls at an early stage. Finally, we used logistic regression to train a model to distinguish patients with late-stage RCC from control subjects without cancer, showing a highest test set sensitivity and specificity of 90% and 92.7%, respectively. These results highlight the strongest dysregulations in sulfatide and sphingolipid levels within this group compared to controls.

Sphingolipid metabolism shows an imbalance in many cancers, which is consistent with the involvement of these lipid classes in basic cellular mechanisms, including proliferation, migration, and senescence [9,28]. Recently, we have reported significant concentration changes of sulfatides in RCC tumor tissues compared to the nonneoplastic tissue part. One of the most important alterations, the downregulation of hydroxylated sulfatides, is observed in all three types of biological matrices in the present study. The structure of the sphingoid base in sulfatides is generally predominantly represented by sphingosine C18, while the N-acyl chain exhibits a greater degree of structural variability in both the length and the range of metabolic modifications, such as desaturation or hydroxylation [29]. The constituents of one of the typical modified N-acyl types are 2-hydroxy fatty acids (hFA), from which the corresponding hFA-sphingolipids are formed. The precursors of hFA-sphingolipids, that is, hydroxy fatty acids (hFAs), are enzymatically formed from fatty acids catalyzed by fatty acid 2-hydroxylase (FA2H, EC 1.14.18.6) [30]. The decrease in FA2H expression and associated reduced levels of hFA-sphingolipids have been described in aggressive and treatment-resistant tumors. Altered FA2H expression may be associated with resistance to treatment [31] and the progression of tumor growth, for example, in lung [32], ovarian [33], colorectal [34], and breast cancer [35]. In turn, increased expression of FA2H and higher levels of hFA-sphingolipids have been described in chemosensitive tumors and patients with a better prognosis [31,33]. Therefore, we hypothesize that determining hydroxylated sulfatides (SHexCer XX:XX;O3 and SHexCer XX:XX;O4) in plasma and urine could potentially have a diagnostic value in RCC as well.

Elevated sulfatide content, especially lactosylsulfatides (SHex2Cer XX:XX;O2) in tumor tissues and urine from RCC patients, is another crucial dysregulation, and might be related to the metabolism of glutamine. Increased glutamine uptake and its utilization in the TCA cycle or as a nitrogen and carbon donor have been observed in many cancers [36], specifically in RCC [37,38]. The dysregulation of glutamine metabolism increases the secretion of ammonium ions. In effect, levels of sulfatides may be influenced, as sulfatides are believed to be involved in renal ammonium handling, urinary acidification, and acid–base homeostasis [39]. This mechanism was already proposed in chronic metabolic acidosis, where renal sulfatides serve the same purpose [39,40].

Concerning sterol sulfates in urine samples, the upregulation of StS 3 and StS 4 (Figure S5) corresponding to elemental compositions of cortisol metabolites might indicate increased stress in patients with RCC [41]. In contrast, the downregulation is observed for sterols StS 8 and StS 11 with the elemental composition corresponding to lithocholic acid metabolites. As lithocholic acid has recently been shown to induce apoptosis within several tumor cell lines [42], its suppression may result from cancer metabolic reprogramming.

Another class of sphingolipids investigated in our work is SM, which are reduced in the tumor tissue and the plasma of RCC patients. For example, SM 41:1;O2 and SM 42:1;O2 are downregulated in both sample matrices, while lower levels of multiple other SM are observed in plasma rather than in the tumor tissue. Reduced levels of SM in RCC have been documented before [11,43], and these findings suggest that SM levels and alteration of the synthetic and degradative pathways play an essential role in cancer. It was reported that SM accumulate in the HCT-116 (human colon cancer cell lines) during apoptosis [44]. Hence, their overall downregulation might be related to the regulation of cancer cell survival [45].

In the majority of cases, the renal mass is detected incidentally during investigations performed by non-invasive imaging methods such as abdominal ultrasound (US) or CT for complaints unrelated to cancer. While abdominal US is more common and available, the sensitivity and specificity of CT in detecting RCC is higher, especially in patients with early-stage tumors. The tumor mass appears solid or cystic, and the most important criterion differentiating malignant lesions from benign is the presence of an enhancement [46]. In case of contraindication of intravenous CT contrast administration, magnetic resonance imaging (MRI) is indicated. However, CT or MRI cannot be recommended for population screening due to cost, radiation exposure, and high potential for additional incidental findings. In turn, US accuracy depends on tumor size, and potential biomarkers in sample matrices such as plasma or urine might represent another indicator of tumor presence that could potentially be taken into consideration while diagnosing cancer. Therefore, we consider the profiling of SM and SHexCer in body fluids as a possible complementary method that may deliver additional valuable clues in the diagnostic process of RCC. However, prospective clinical validation on the larger cohort has to be performed first in order to prove the real clinical utility of this methodology.

## 5. Conclusions

The key outcome of our research is the discovery of significantly changed profiles of sulfatides and sphingomyelins in plasma and urine taken from RCC patients compared to controls without cancer. We found that lipid concentrations in body fluids and tissues show similar trends in lipid dysregulations and that the observed alterations are very likely to be associated with the progression of RCC. Our results demonstrate that RCC widely influences lipid metabolism, highlighting the potential of the studied lipids for effective and minimally-invasive cancer detection, including in patients with early tumor stages, as demonstrated by the predictive ability of the applied classification models.

## Data Availability

All data necessary to support the conclusions are available in the manuscript or Supplementary Materials. Raw data, instructions for data processing, and Excel macro script are available from the online open-access repository Figshare at https://figshare.com/s/d01d43e862b7e03adbfe.

## Supplementary Materials

The following supporting information can be downloaded at: https://www.mdpi.com/article/10.3390/cancers14194622/s1, Figure S1: PCA-X score plots and volcano plots built based on the evaluation of relative concentrations of studied lipids in plasma, urine, and tissue samples; Figure S2: Radar charts of lipids analyzed using the present/absent approach for plasma and urine samples of controls and RCC patients; Figure S3: Box plots illustrating differences between controls and patients with different RCC subtypes in relative concentrations of selected sphingomyelins and sulfatides in plasma samples; Figure S4: Cytoscape networks illustrating differences between the results of the Mann-Whitney U test and fold change analysis in two data sets obtained for urine lipidome profiling: for the data set with 27 subjects excluded (A) and for the whole urine data set without any exclusions (B). Figure S5: Box plots of relative concentrations of selected sterols measured in urine samples for controls and patients with RCC; Figure S6: Comparison of the most statistically significant lipids in tumor tissue and surrounding nonneoplastic tissue of RCC patients with early tumor stage (T1-T2) and RCC patients with more advanced tumor stage (T3-T4); Figure S7: Box plots showing differences in relative concentrations of selected lipids between RCC patients with T3 tumor stage with or without metastases, and box plots showing subtle differences in the relative concentrations of selected lipids between genders in studied plasma samples; Data S1: Clinical data together with raw intensities, relative and molar concentrations of individual lipids measured in studied plasma, urine, and tissue samples of RCC patients and healthy controls; Data S2: Statistical calculations supporting the article “Altered plasma, urine, and tissue profiles of sulfatides and sphingomyelins in patients with renal cell carcinoma”; Data S3: Lipid annotation, analytical validation, and quality control within the article: Altered plasma, urine, and tissue profiles of sulfatides and sphingomyelins in patients with renal cell carcinoma. References [47,48,49,50,51] are cited in Supplementary Materials.
