# Supplementary material for: Altered Plasma, Urine, and Tissue Profiles of Sulfatides and Sphingomyelins in Patients with Renal Cell Carcinoma
Source: Cancers (Basel). 2022 Sep 23;14(19):4622. doi: 10.3390/cancers14194622 (PMC9563753; doi:10.3390/cancers14194622)
Supplement: Supplementary file 1 [file cancers-14-04622-s001.zip › cancers-1894222-supplementary/Supplementary_Data_S3__Cancers.pdf]

Supplementary Data S3

Lipid annotation, analytical validation, and quality control within the article:

Altered plasma, urine, and tissue profiles of sulfatides and sphingomyelins in patients with renal cell carcinoma

Robert Jirásko[1*], Jakub Idkowiak[1], Denise Wolrab[1], Aleš Kvasnička[2], David Friedecký[2], Krzysztof Polański[3], Hana Študentová[4], Vladimír Študent[5], Bohuslav Melichar[4] and Michal Holčapek[1]

[1]Department of Analytical Chemistry, Faculty of Chemical Technology, University of Pardubice, Studentská 573, 53210 Pardubice, Czech Republic

[2]Laboratory for Inherited Metabolic Disorders, Faculty of Medicine and Dentistry, Palacký University and Department of Clinical Biochemistry, University Hospital Olomouc, 779 00 Olomouc, Czech Republic

[3]Wellcome Sanger Inst, Wellcome Genome Campus, Cambridge, England

[4]*Department of Oncology, Faculty of Medicine and Dentistry, Palacký University and University Hospital, Olomouc, Czech Republic*

[5]*Department of Urology, Faculty of Medicine and Dentistry, Palacký University and University Hospital, Olomouc, Czech Republic*

Corresponding author:

Robert Jirásko, Ph.D., Department of Analytical Chemistry, Faculty of Chemical Technology, University of Pardubice, Studentská 573, 53210 Pardubice, Czech Republic, e-mail: robert.jirasko@upce.cz, phone: +420 466 037 089, fax: +420 466 037 068

Content

Lipid annotation 2
Method validation 2
Quality control and NIST plasma measurement 4
References 5
Figure S1 6
Figure S2 7
Figure S3 8
Figure S4 9
Table S1 10
Table S2 11
Table S3 12
Table S4 13
Table S5 14
Table S6 15
Table S7 16
Table S8 17
Table S9 18
Table S10 19

Lipid annotation

The lipid nomenclature followed LIPID MAPS system [25] and the shorthand notation for lipid structures [26,27]. In the following text, sulfatide shorthand notification is briefly described on the example of sulfatide SHex2Cer 34:1;O2 for a better understanding. First capital letter S means presence of one sulfate group, the abbreviation Hex means the presence of hexosyl unit without specification of stereochemistry, and the additional number gives the information on their number (in our example, two hexosyl units) on ceramide backbone. Subsequently, the colon-separated numbers 34:1 provides the information on the total number of carbon atoms and double bonds (CN:DB) of N-linked fatty acyl and sphingoid base of ceramide part, and a semicolon separated O2 informs about two hydroxyl oxygens in the ceramide part without any specification of their position. In the case that an additional hydroxyl is present in the ceramide part without any specification of its position O3 is written behind semicolon (in case of two additional hydroxyls - O4).

For sterol sulfates, we have been able to verify the presence of the sulfate group according to MS/MS spectra, where a typical neutral loss of 80 was observed, and using a high-resolution mass spectra measurement that allowed us to determine the elemental composition. However, the exact structure elucidation, including the isomerism of sterols, is not possible only by using MALDI-Orbitrap MS measurement. Several sterol isomers with identical elemental composition can contribute to the ion signal. For this reason, we have abbreviated the observed sterols from StS 1 to StS 13 and we only suggested the most probable compounds for individual elemental compositions in Figure S3A.

Method validation

All used internal standards (SHexCer 18:1;O2/12:0, SM d18:1;O2/12:0, and taurocholic acid-d4) were tested for their applicability for quantitation of plasma or urine samples. The bioanalytical method validation guidelines [47,48] were followed and adapted. Representative samples of controls and cases characterized by different gender, age, and health state, were mixed in equal ratios for the pooled sample preparation - QC. The parameters determined included calibration curve, selectivity, repeatability, extraction recovery, matrix effect, within-run and between-run precision, carry-over effect, and freeze/thaw stability. Aliquots of the pooled sample were either not spiked, spiked before, or after extraction with a mixture of IS at a low, medium, or high concentration level, respectively. Medium

concentration was identical to the IS concentrations in studied plasma and urine samples of controls and RCC patients. Individual IS concentration in plasma and urine are listed in Table V1.

The reproducibility of sample spotting is represented by the coefficients of variation (CV) calculated from five IS intensities of 5 consecutive spots of each sample (Tables V2-V4). All validation parameters were calculated using Microsoft Excel® 2016 (ver. 2102). The investigated parameters were determined on two independent days (except freeze/thaw stability). The linearity range and the limit of quantitation (LOQ) of the developed MALDI-MS method were obtained from a calibration curve based on the extracted pooled sample of plasma or urine spiked with IS mixture at different concentration levels (Table V5 and Figure V1a-d). Calibration curves were plotted as peak abundances against concentrations of IS individual calibration solutions at 8 concentration levels and fitted by linear regression. The measurement of high number of samples with five repetitions is not possible within one run on one MALDI plate. The washing step of the plate is necessary before the next run, and the carry-over effect should be determined to verify the efficiency of the washing procedure. The carry-over effect was determined from the blank sample, which was spotted on the washed MALDI plate on the same positions as previously measured calibration standards at the upper limit of quantitation. No signal at m/z corresponding applied IS was observed for both sample types. The extraction recovery was determined by calculating the ratio of the IS signal intensity of 6 samples (3 for urine) spiked before and 6 samples (3 for urine) spiked after the extraction at three concentration levels (Tables V6, Table V9, and Figure V1e, f). Within-run precision (Table V8) was calculated as the CV of IS intensities (IS spiked before extraction) within three consecutive extractions performed in one day for low, medium (only for plasma), and high concentration levels. The between-run precision (Table V8) was determined based on the measurement of six extracts of the pooled samples at three concentration levels (three extracts were prepared in one day, and another three extracts were prepared in the second day). For evaluating selectivity, matrix effect, and freeze/thaw stability of the plasma samples, plasma extracts of six different individuals were measured (Table V3). The selectivity of the tested IS was verified based on the evaluation of the data obtained after measurement of these samples without the addition of IS, where no interference signal was observed at *m/z* corresponding to IS. The matrix effect was calculated as the coefficient of variation of IS intensities within the mass spectra of samples of six different individuals

at three concentration levels (Table V9). Freeze/thaw stability was determined individually for each sample of 6 individuals with IS at the medium level, and it was calculated as the coefficient of variation of four median intensities within three freeze/thaw cycles involving the storage of the sample at -20°C overnight and its thawing at room temperature for 1 h (Table V10). For urine samples, the selectivity of the tested IS was verified based on the evaluation of data obtained after measurement of pooled urine and urine samples of six random individuals (without the addition of IS), where no signal at m/z of IS was observed in all cases. Freeze/thaw stability of urine was verified based on the comparison of IS intensities of the pooled sample with IS at low and high concentration levels after one freeze/thaw cycle involving the storage of the sample at -20°C overnight and its thawing at room temperature for 1 h (Table V10). High matrix effect was observed for urine samples as evident from the Supplementary Data 2 (raw intensities) of IS intensities in the studied samples resulting in the decision to normalize the data to the sum of all intensities for all lipid species within a particular class for each sample separately to calculate relative concentrations in %.

Quality control and NIST plasma measurement

Pooled samples were used as quality control (QC) to monitor the instrument performance and the quality of data obtained in sequence measurement of plasma and urine samples. At least 3 QC samples were spotted on each MALDI plate at the beginning, middle, and end of the sequence measurement. Figures V2 and V3 show the variation in absolute intensities of IS and selected endogenously present lipids within all measured QC samples. The middle line represents average intensity, and the dashed lines represent the coefficient of variation of 20%. The coefficient of variation of the absolute signal, which in some cases exceeds 20%, especially for SHexCer 41:1;O3, is reduced by normalization to IS or sum of intensities of all quantified sulfatides (relative concentrations), which is shown in Figures V2d and V13f, where the absolute concentration of selected lipids within QC samples are replaced by molar or relative concentrations. Similarly, for urine samples, the coefficient of variation is reduced in the case of monitoring relative concentrations (Figures V3). The clusters of QC samples in the PCA score plots indicated method stability during measurements (see Supplementary Fig. S1). Another verification of the reliability of our semiquantitative method was performed using the measurement of NIST plasma as available standard reference materials to compare the obtained concentrations with literature [49-51].

The measurement of NIST plasma shows comparable results to previously published data (Figure V4). Unfortunately, no information about sulfatide concentrations is found in the literature and the comparison of obtained concentrations is performed only for SM lipid class.

References

(reference numbers correspond to numbers in the main part of the manuscript)

25. Lipid Maps. Lipid Classsification System. Available online: https://www.lipidmaps.org/data/classification/LM_classification_exp.php (accessed on 29 July 2022).

26. Liebisch, G.; Fahy, E.; Aoki, J.; Dennis, E.A.; Durand, T.; Ejsing, C.S.; Fedorova, M.; Feussner, I.; Griffiths, W.J.; Kofeler, H.; et al. Update on LIPID MAPS classification, nomenclature, and shorthand notation for MS-derived lipid structures. *J. Lipid Res.* **2020**, *61*, 1539-1555, doi:10.1194/jlr.S120001025.

27. Liebisch, G.; Vizcaino, J.A.; Kofeler, H.; Trotzmuller, M.; Griffiths, W.J.; Schmitz, G.; Spener, F.; Wakelam, M.J.O. Shorthand notation for lipid structures derived from mass spectrometry. *J. Lipid Res.* **2013**, *54*, 1523-1530, doi:10.1194/jlr.M033506.

47. European Medicines Agency, Guideline on bioanalytical method validation,Committee for Medicinal Products for Human Use, 192217/2009, Rev. 1 Corr.2, 21 July 2011. *European Medicines Agency, Guideline on bioanalytical method validation,Committee for Medicinal Products for Human Use, 192217/2009, Rev. 1 Corr.2, 21 July 2011.*

48. Food and Drug Administration, Guidance for Industry on BioloanalyticalMethod Validation, Federal Register, 23 May 2001. *Food and Drug Administration, Guidance for Industry on BioloanalyticalMethod Validation, Federal Register, 23 May 2001.*

49. Bowden, J.A.; Heckert, A.; Ulmer, C.Z.; Jones, C.M.; Koelmel, J.P.; Abdullah, L.; Ahonen, L.; Alnouti, Y.; Armando, A.M.; Asara, J.M.; et al. Harmonizing lipidomics: NIST interlaboratory comparison exercise for lipidomics using SRM 1950–Metabolites in Frozen Human Plasma[S]. *J. Lipid Res.* **2017**, *58*, 2275-2288, doi:https://doi.org/10.1194/jlr.M079012.

50. Huynh, K.; Barlow, C.K.; Jayawardana, K.S.; Weir, J.M.; Mellett, N.A.; Cinel, M.; Magliano, D.J.; Shaw, J.E.; Drew, B.G.; Meikle, P.J. High-Throughput Plasma Lipidomics: Detailed Mapping of the Associations with Cardiometabolic Risk Factors. *Cell Chem. Biol.* **2019**, *26*, 71-84, doi:https://doi.org/10.1016/j.chembiol.2018.10.008.

51. Quehenberger, O.; Armando, A.M.; Brown, A.H.; Milne, S.B.; Myers, D.S.; Merrill, A.H.; Bandyopadhyay, S.; Jones, K.N.; Kelly, S.; Shaner, R.L.; et al. Lipidomics reveals a remarkable diversity of lipids in human plasma1[S]. *J. Lipid Res.* **2010**, *51*, 3299-3305, doi:https://doi.org/10.1194/jlr.M009449.

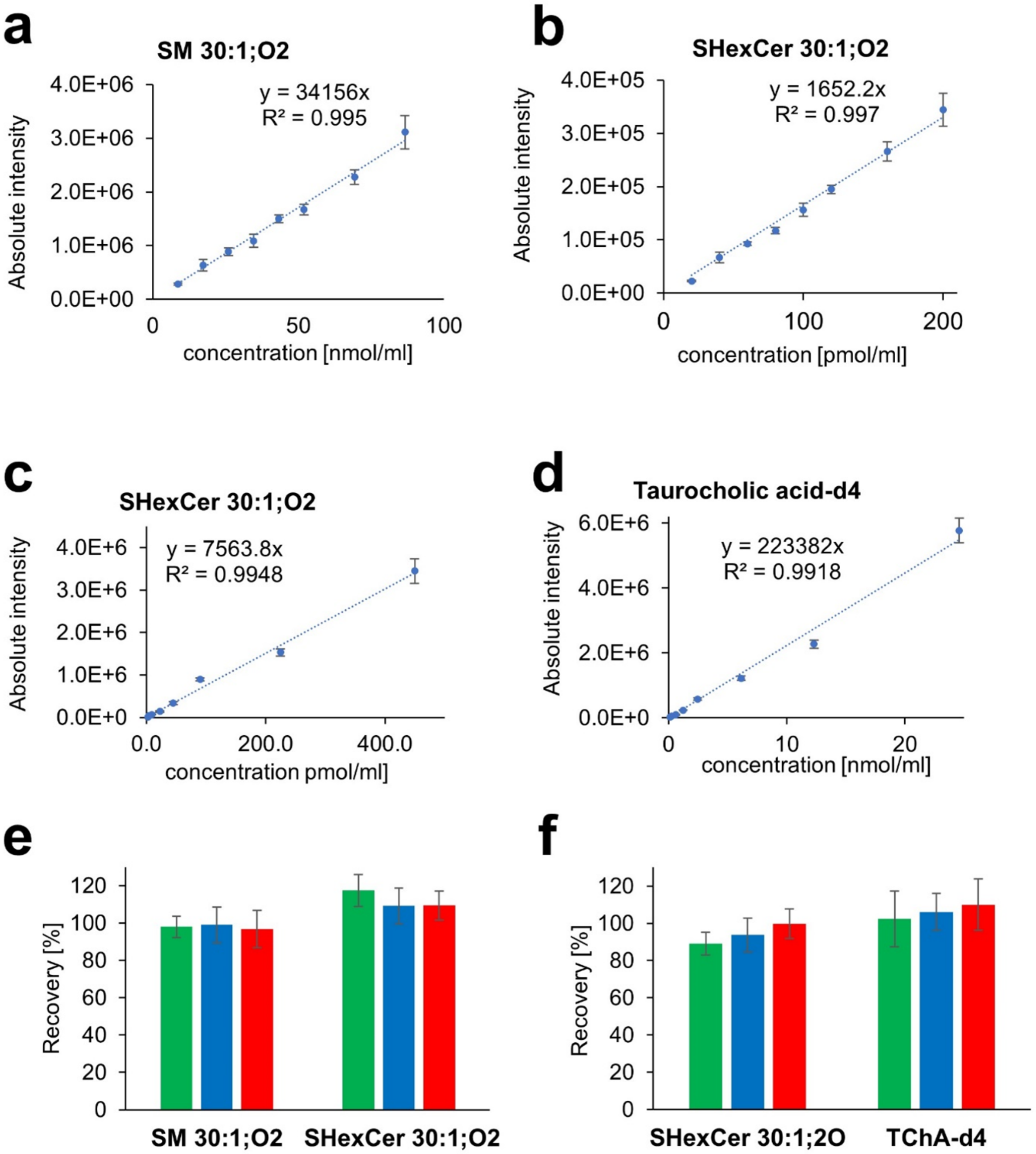

Figure S1 Calibration curves present linear concentration ranges for applied internal standards in (a, b) plasma and (c, d) urine samples (error bars represent SD from five consecutive measurements). Extraction recovery of internal standards used for the extraction of (e) plasma and (f) urine samples (error bars represent the coefficient of variation of 6 independent extractions in the case of plasma and 3 independent extractions in the case of urine).

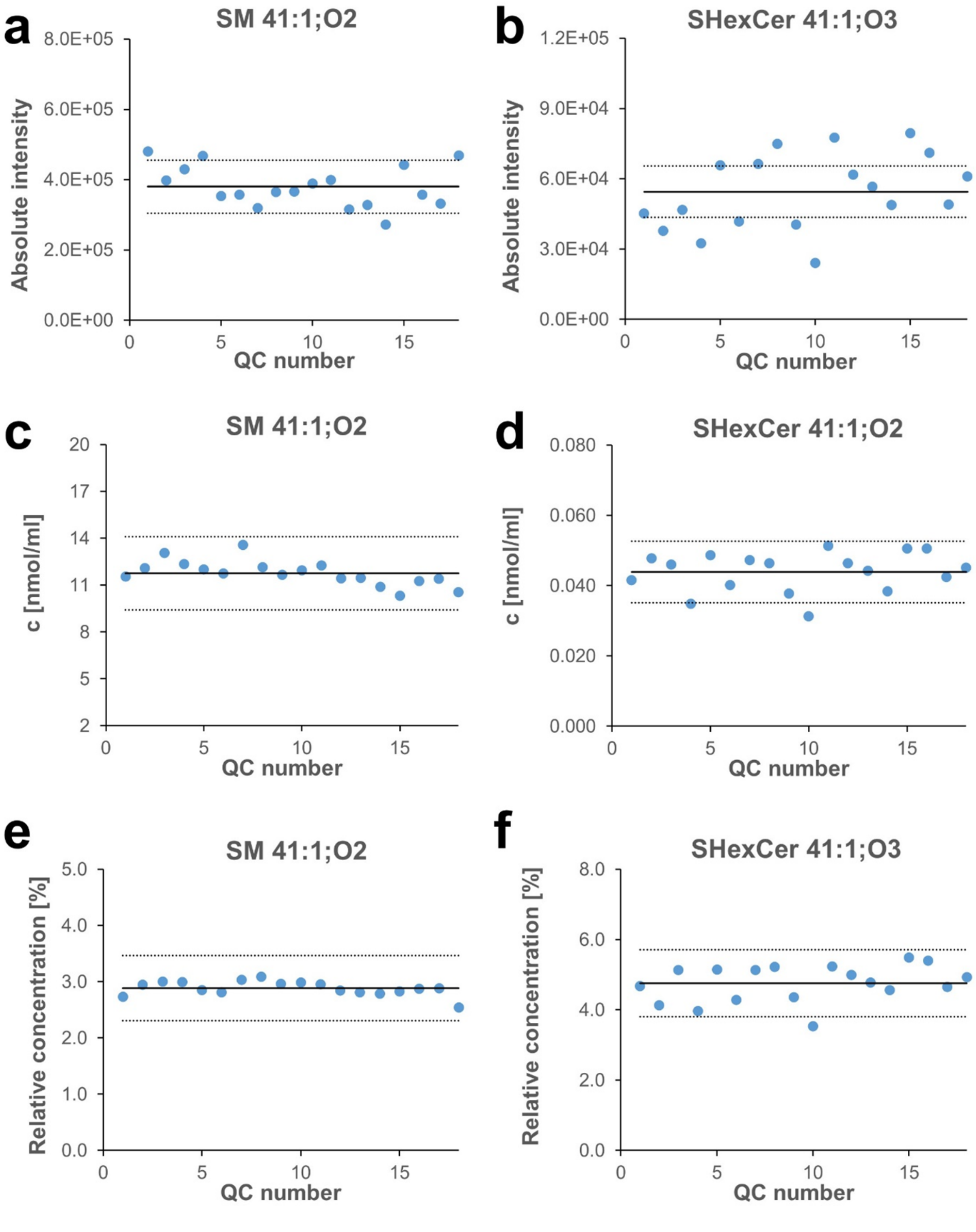

Figure S2 Quality control of lipidomic analysis based on the response of 18 QC samples during the sequence measurement of plasma samples in the example of endogenously present lipids SM 41:1;O2 and SHexCer 41:1;O3: **a, b** absolute intensities; **c, d** calculated molar concentrations; **e, f** relative concentrations. The middle full lines represent the average of individual values (intensities, molar, or relative concentrations), and two dotted lines are twenty percentage deviations from this average value.

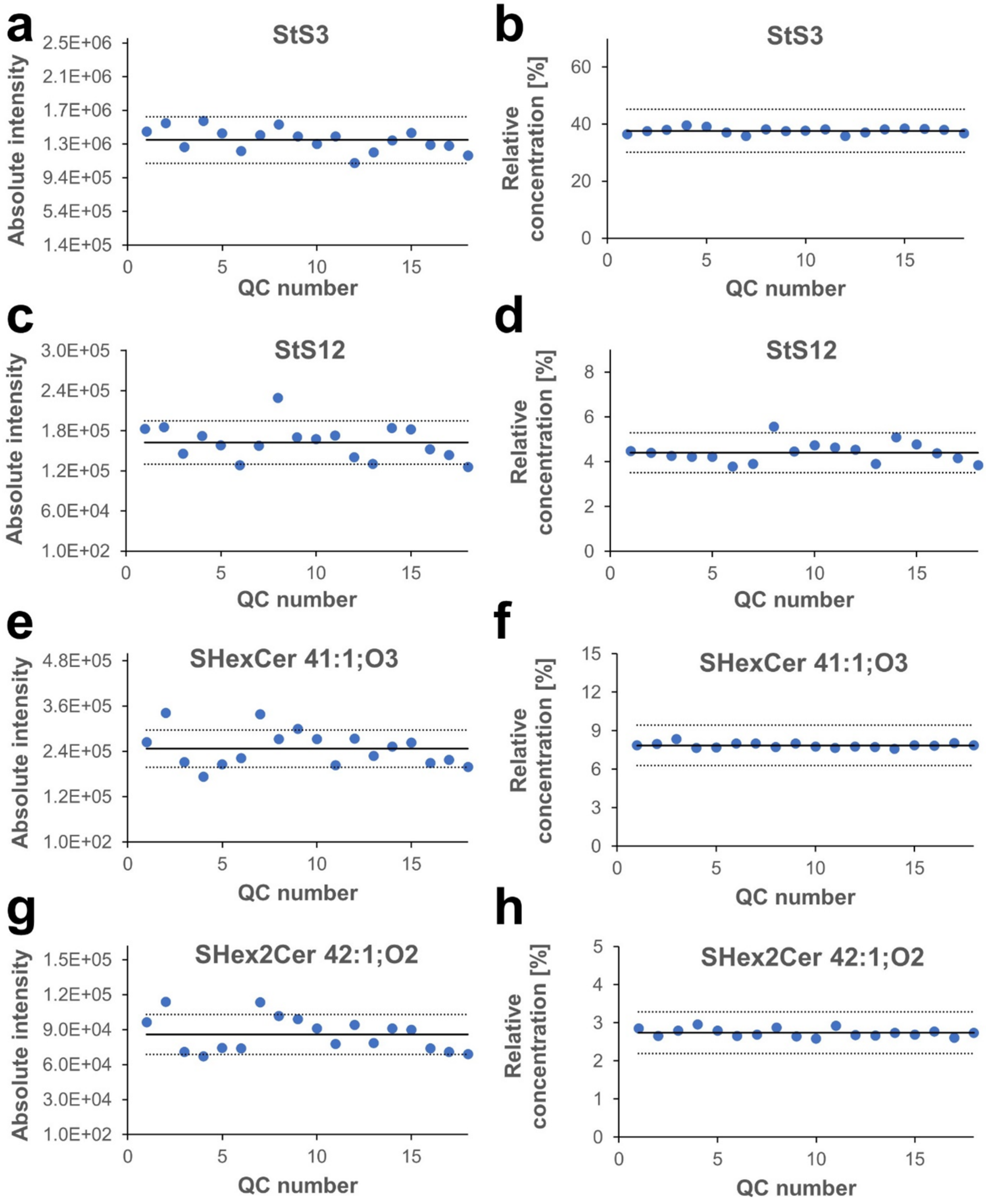

Figure S3 Quality control of lipidomic analysis based on the response signals of QC samples during the sequence measurement of urine samples in the example of endogenously present lipids StS3, StS12, SHexCer 41:1;O3 and SHex2Cer 42:1;O2: **a, c, e, g** absolute intensities; **b, d, f, h** relative concentrations. The middle full lines represent the average of individual values (intensities or relative concentrations), and two dotted lines are twenty percentage deviations from this average value.

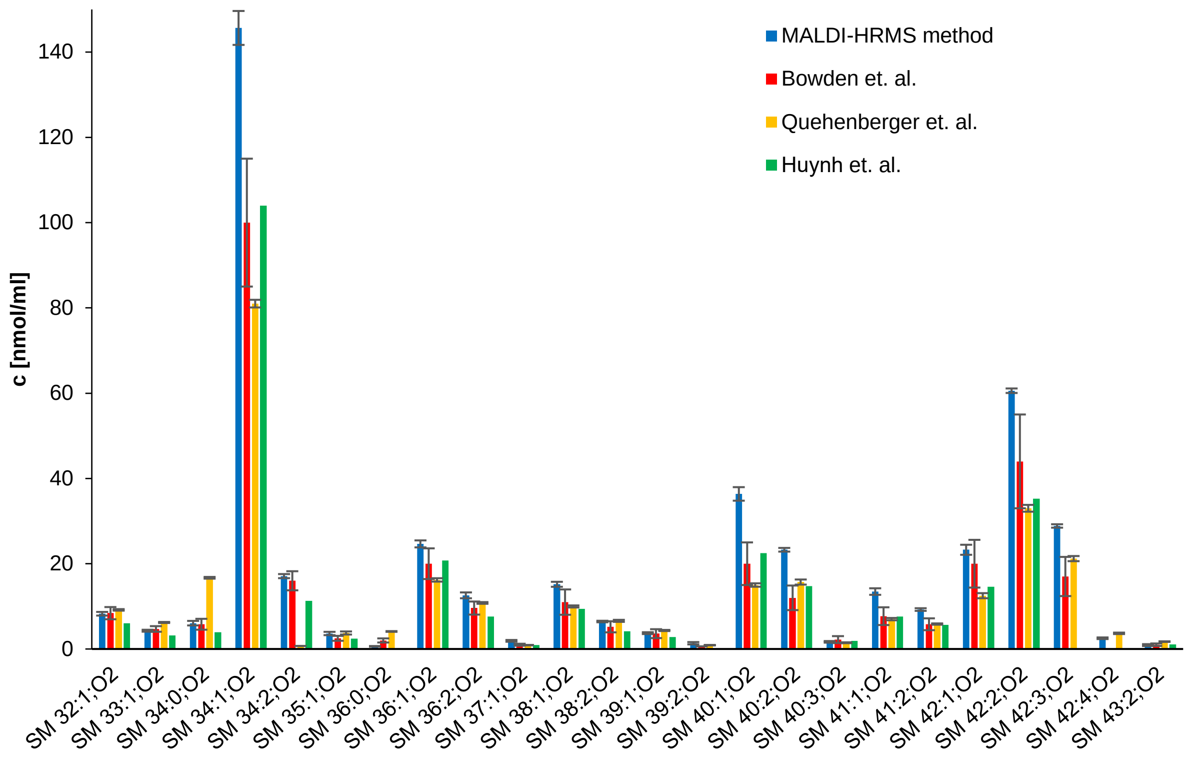

Figure S4 Bar graph compares molar concentrations between our MALDI-HRMS method (blue bars, error bars represent standard deviation (SD) and were calculated based on the three
independently extracted samples) and other data from the literature[49-51].

Table S1 List of concentrations of individual internal standards used for the analytical validation experiments in plasma and urine samples for low (LL), medium (ML), and high (HL) concentration levels.

	**c [nmol/ml of plasma]**		
	**LL**	**ML**	**HL**
SM 18:1;O2/12:0	21.65	43.3	64.95
SHexCer 18:1;O2/12:0	0.05	0.1	0.15

	**c [nmol/ml of urine]**		
	**LL**	**ML**	**HL**
Taurocholic acid D4	0.183	0.55	1.65
SHexCer 18:1;O2/12:0	0.013	0.04	0.12

Table S2 Median of absolute intensities and individual coefficients of variation (CV) for each IS - calculated from 5 repetitions (consecutive measurements) within individual samples of the plasma validation experiment. Data have been used to calculate recovery rates (x = IS added before the extraction and y = IS added after extraction) and precisions (x). The individual CV values represent repeatability.

IS addition	Day	Level	SM 30:1;O2		SHexCer 30:1;O2	
			Median of Intensity	CV [%]	Median of Intensity	CV [%]
Low level (IS added before extraction)	1st day	Lx1	8.25E+05	7	1.00E+05	19
		Lx2	7.62E+05	5	8.94E+04	12
		Lx3	7.57E+05	8	8.15E+04	14
	2nd day	Lx4	7.76E+05	7	1.05E+05	4
		Lx5	8.20E+05	7	9.94E+04	6
		Lx6	8.29E+05	14	1.04E+05	4
Low level (IS added after extraction)	1st day	Ly1	7.83E+05	10	7.72E+04	11
		Ly2	7.45E+05	14	7.36E+04	10
		Ly3	7.65E+05	12	6.74E+04	12
	2nd day	Ly4	8.59E+05	8	1.03E+05	5
		Ly5	9.05E+05	4	7.98E+04	2
		Ly6	8.27E+05	7	9.78E+04	9
Medium level (IS added before extraction)	1st day	Mx1	1.09E+06	5	1.12E+05	16
		Mx2	9.78E+05	14	1.15E+05	10
		Mx3	1.05E+06	8	1.14E+05	2
	2nd day	Mx4	9.10E+05	5	1.34E+05	8
		Mx5	1.13E+06	9	1.36E+05	10
		Mx6	1.03E+06	4	1.38E+05	8
Medium level (IS added after extraction)	1st day	My1	1.09E+06	10	9.04E+04	5
		My2	1.04E+06	10	9.92E+04	11
		My3	9.07E+05	7	9.96E+04	10
	2nd day	My4	1.07E+06	7	1.40E+05	7
		My5	1.08E+06	8	1.42E+05	9
		My6	1.08E+06	5	1.27E+05	9
High level (IS added before extraction)	1st day	Hx1	1.67E+06	6	2.19E+05	5
		Hx2	2.06E+06	14	2.32E+05	9
		Hx3	1.76E+06	17	2.28E+05	16
	2nd day	Hx4	1.71E+06	11	2.44E+05	5
		Hx5	1.91E+06	19	2.42E+05	9
		Hx6	1.77E+06	6	2.36E+05	9
High level (IS added after extraction)	1st day	Hy1	1.92E+06	18	1.97E+05	17
		Hy2	1.78E+06	12	1.99E+05	7
		Hy3	1.90E+06	12	2.00E+05	20
	2nd day	Hy4	1.90E+06	7	2.05E+05	5
		Hy5	1.86E+06	10	2.36E+05	14
		Hy6	1.92E+06	10	2.52E+05	6

Table S3 Median of absolute intensities and individual coefficients of variation (CV) for each IS - calculated from 5 repetitions (consecutive measurements) within individual samples of plasma validation experiments. Data obtained for samples "before freeze/thaw" were used for matrix effect evaluation at three concentration levels. The freeze/thaw stability was evaluated based on the three freeze/thaw cycles for the medium concentration level. The individual CV values represent repeatability.

Concentration level	Cycle	Sample No.	SM 30:1;O2		SHexCer 30:1;O2	
			Median of Intensity	CV [%]	Median of Intensity	CV [%]
Low level	before freeze/thaw	L_31	9.08E+05	3	9.42E+04	9
		L_43	8.57E+05	8	8.69E+04	11
		L_96	8.00E+05	7	8.26E+04	9
		L_97	9.50E+05	12	8.15E+04	8
		L_101	1.17E+06	18	8.78E+04	5
		L_107	9.13E+05	6	7.09E+04	13
High level	before freeze/thaw	H_31	1.81E+06	9	2.24E+05	2
		H_43	1.66E+06	6	1.68E+05	11
		H_96	1.51E+06	8	1.67E+05	4
		H_97	1.94E+06	6	1.82E+05	17
		H_101	2.49E+06	9	2.28E+05	8
		H_107	2.03E+06	5	1.99E+05	6
Medium level	before freeze/thaw	M_31	1.35E+06	4	1.34E+05	7
		M_43	1.01E+06	5	1.06E+05	9
		M_96	1.04E+06	11	1.05E+05	13
		M_97	1.02E+06	9	1.08E+05	16
		M_101	1.40E+06	11	1.11E+05	3
		M_107	1.14E+06	5	8.73E+04	5
	1st freeze/thaw cycle	M_31	1.03E+06	8	9.81E+04	3
		M_43	8.94E+05	8	9.35E+04	17
		M_96	8.52E+05	9	8.43E+04	3
		M_97	8.49E+05	12	8.38E+04	14
		M_101	1.14E+06	10	8.62E+04	6
		M_107	1.29E+06	9	9.62E+04	21
	2nd freeze/thaw cycle	M_31	8.81E+05	18	8.92E+04	14
		M_43	8.96E+05	10	7.19E+04	22
		M_96	1.10E+06	6	1.09E+05	14
		M_97	9.27E+05	7	8.61E+04	16
		M_101	1.09E+06	9	7.96E+04	23
		M_107	1.17E+06	9	1.04E+05	23
	3rd freeze/thaw cycle	M_31	8.83E+05	5	8.98E+04	10
		M_43	1.33E+06	13	1.42E+05	8
		M_96	8.63E+05	4	7.86E+04	11
		M_97	7.78E+05	11	6.87E+04	20
		M_101	1.03E+06	3	5.67E+04	18
		M_107	8.44E+05	7	6.88E+04	10

Table S4 Median of absolute intensities and individual coefficients of variation (CV) for IS used in the case of urine samples - calculated from 5 repetitions within the individual sample type of urine validation experiment. Data have been used for the calculation of recovery rates (x = IS added before the extraction) and y = IS added after extraction), precisions (x), and freeze/thaw stability. The individual CV values represent repeatability.

Concentration level (IS addition)	Day	Level	SHexCer 30:1;O2		Taurocholic acid-d4	
			Median of Intensity	CV [%]	Median of Intensity	CV [%]
Low level (IS added before extraction)	1st day	Lx1	2.72E+04	16	1.84E+04	4
		Lx2	2.43E+04	24	1.64E+04	7
		Lx3	3.42E+04	12	1.28E+04	3
	2nd day	Lx4	2.67E+04	21	1.81E+04	22
		Lx5	2.57E+04	14	1.98E+04	21
		Lx6	1.55E+04	23	9.80E+03	34
Low level (IS added after extraction)	1st day	Ly1	3.20E+04	10	1.55E+04	7
		Ly2	2.88E+04	13	1.74E+04	8
		Ly3	3.50E+04	14	1.49E+04	11
Medium level (IS added before extraction)	1st day	Mx1	1.06E+05	9	6.60E+04	3
		Mx2	7.54E+04	15	5.38E+04	16
		Mx3	9.22E+04	12	7.86E+04	3
Medium level (IS added after extraction)	1st day	My1	1.01E+05	19	7.64E+04	11
		My2	9.10E+04	9	5.51E+04	12
		My3	9.89E+04	13	6.55E+04	8
High level (IS added before extraction)	1st day	Hx1	4.83E+05	19	3.15E+05	2
		Hx2	5.52E+05	5	3.03E+05	4
		Hx3	4.73E+05	15	2.98E+05	11
	2nd day	Hx4	5.05E+05	16	2.71E+05	13
		Hx5	5.44E+05	13	2.46E+05	10
		Hx6	5.11E+05	11	3.28E+05	14
High level (IS added after extraction)	1st day	Hy1	3.87E+05	12	2.40E+05	13
		Hy2	3.37E+05	12	1.97E+05	5
		Hy3	3.72E+05	21	2.78E+05	5
freeze/thaw cycle	3rd day	Lx4	3.25E+04	20	1.30E+04	16
		Lx5	2.74E+04	24	1.29E+04	12
		Lx6	1.75E+04	33	9.91E+03	30
freeze/thaw cycle	3rd day	Hx4	3.42E+05	15	1.82E+05	10
		Hx5	3.48E+05	14	2.20E+05	5
		Hx6	2.80E+05	11	2.55E+05	6

Table S5 Raw IS intensities and individual coefficients of variation (n=5) used for calibration curves - for plasma (upper part of the table) and urine samples (bottom part of the table).

SM 30:1;O2			SHexCer 30:1;O2		
c [nmol/mL]	Median of Intensity	CV[%]	c [pmol/mL]	Median of Intensity	CV [%]
8.66	2.80E+05	6	20	2.23E+04	5
17.32	6.35E+05	17	40	6.67E+04	15
25.99	8.90E+05	8	60	9.26E+04	3
34.65	1.09E+06	11	80	1.17E+05	5
43.31	1.50E+06	5	100	1.56E+05	8
51.97	1.67E+06	6	120	1.95E+05	4
69.3	2.28E+06	6	160	2.66E+05	7
86.62	3.11E+06	10	200	3.44E+05	9

SHexCer 30:1;O2			Taurocholic acid-d4		
c [pmol/mL]	Median of Intensity	CV [%]	c [nmol/mL]	Median of Intensity	CV [%]
2.25	1.13E+04	13	0.12	1.65E+04	10
4.50	4.29E+04	20	0.25	5.84E+04	7
8.99	6.93E+04	10	0.62	9.73E+04	11
22.49	1.38E+05	11	1.23	2.38E+05	5
44.97	3.35E+05	10	2.46	5.80E+05	6
89.94	8.95E+05	4	6.15	1.22E+06	5
224.85	1.53E+06	6	12.30	2.27E+06	5
449.71	3.45E+06	9	24.61	5.77E+06	7

Table S6 Individual recovery rates calculated based on the raw data in Supplementary Table 5 together with average values and coefficients of variation obtained for the extraction protocol of pooled plasma sample for applied internal standards, where abbreviation LL means low concentration level, ML means medium concentration level, and HL means high concentration level.

Level	Sample No.	Recovery rate [%]	
		SM 30:1;O2	SHexCer 30:1;O2
Low level	LL1	105	130
	LL2	102	121
	LL3	99	121
	LL4	90	101
	LL5	91	125
	LL6	100	107
	Average	98	118
	CV [%]	6	9
Medium level	ML1	100	124
	ML2	94	116
	ML3	115	114
	ML4	85	96
	ML5	105	96
	ML6	95	109
	Average	99	109
	CV [%]	10	10
High level	HL1	87	111
	HL2	115	116
	HL3	93	114
	HL4	90	119
	HL5	103	103
	HL6	92	94
	Average	97	109
	CV [%]	10	8

Table S7 Individual recovery rates calculated based on the raw data in Supplementary Table 7 together with average values and coefficients of variation obtained for the extraction protocol of the pooled urine sample for applied internal standards, where abbreviation LL means low concentration level, ML means medium concentration level, and HL means high concentration level.

Level	Sample No.	Recovery rate [%]	
		SHexCer 30:1;O2	Taurocholic acid-d4
Low level	LL1	85	119
	LL2	84	106
	LL3	98	83
	Average	89	102
	CV [%]	6	15
Medium level	ML1	105	101
	ML2	83	98
	ML3	93	120
	Average	94	106
	CV [%]	9	10
High level	HL1	96	116
	HL2	111	123
	HL3	93	91
	Average	100	110
	CV [%]	8	14

Table S8 Coefficients of variation representing within (n=3) and between (n=6) days precision for internal standards applied for plasma measurements (upper part of the table) and urine measurements (bottom part of the table). Individual average intensities and CV were calculated from the absolute intensities of Supplementary Tables 5 (plasma) and 7 (urine). The abbreviation LL means low concentration level, ML means medium concentration level and HL means high concentration level.

Type	Level	SM 30:1;O2		SHexCer 30:1;O2	
		Average Intensity	CV [%]	Average Intensity	CV [%]
Within day presicion	LL	7.81E+05	4	9.04E+04	9
	ML	1.04E+06	4	1.14E+05	1
	HL	1.83E+06	9	2.26E+05	2
Between day precision	LL	7.95E+05	4	9.66E+04	9
	ML	1.03E+06	7	1.25E+05	9
	HL	1.80E+06	7	2.41E+05	4

Type	Level	SM 30:1;O2		SHexCer 30:1;O2	
		Average Intensity	CV [%]	Average Intensity	CV [%]
Within day presicion	LL	2.86E+04	15	1.59E+04	14.6
	HL	5.03E+05	7	3.05E+05	2.3
Between day precision	LL	2.56E+04	22	1.59E+04	22.0
	HL	4.34E+05	17	2.72E+05	15.1

Table S9 Matrix effect of internal standards used for plasma measurements. The matrix effect is expressed by the coefficient of variation of average signal response within 6 different plasma samples (individual raw intensities are shown in Supplementary Table 6) at three concentration levels, where the abbreviation LL means low concentration level, ML means medium concentration level, and HL means high concentration level.

Level	SM 30:1;O2		SHexCer 30:1;O2	
	Average Intensity	CV [%]	Average Intensity	CV [%]
LL	9.33E+05	12	8.40E+04	9
ML	1.16E+06	14	1.08E+05	13
HL	1.91E+06	16	1.95E+05	13

Table S10 The upper part of the table shows the freeze/thaw stability of internal standards at medium concentration level applied for plasma measurements. Freeze/thaw stability is expressed by the coefficient of variation of the average signal response for 6 individual plasma samples after 3 freeze/thaw cycles (individual raw intensities are shown in Supplementary Table 6). The bottom part of the table shows freeze/thaw stability of internal standards applied for urine measurements. The freeze/thaw stability is expressed by the coefficient of variation (n=3) of average signal response for the pooled sample at a low (LL) and high (HL) concentration levels after one freeze/thaw cycle (individual absolute intensities of LL4 – LL6 and HL4 – HL6 before and after freeze/thaw cycle are shown in Supplementary Table 8).

Sample No	SM 30:1;O2		SHexCer 30:1;O2	
	Average Intensity	CV [%]	Average Intensity	CV [%]
M_31	1.04E+06	18	1.03E+05	18
M_43	1.03E+06	17	1.03E+05	25
M_96	9.65E+05	11	9.41E+04	14
M_97	8.93E+05	10	8.66E+04	16
M_101	1.17E+06	12	8.35E+04	23

Level	Taurocholic acid-d4		SHexCer 30:1;O2	
	Average Intensity	CV [%]	Average Intensity	CV [%]
LL	1.39E+04	27	2.42E+04	24.4
HL	2.29E+05	14.5	3.44E+05	9.8
