# Supplementary material for: Altered Plasma, Urine, and Tissue Profiles of Sulfatides and Sphingomyelins in Patients with Renal Cell Carcinoma
Source: Cancers (Basel). 2022 Sep 23;14(19):4622. doi: 10.3390/cancers14194622 (PMC9563753; doi:10.3390/cancers14194622)
Supplement: Supplementary file 1 [file cancers-14-04622-s001.zip › cancers-1894222-supplementary/Supplementary_FIGURES_S1-S7.pdf]

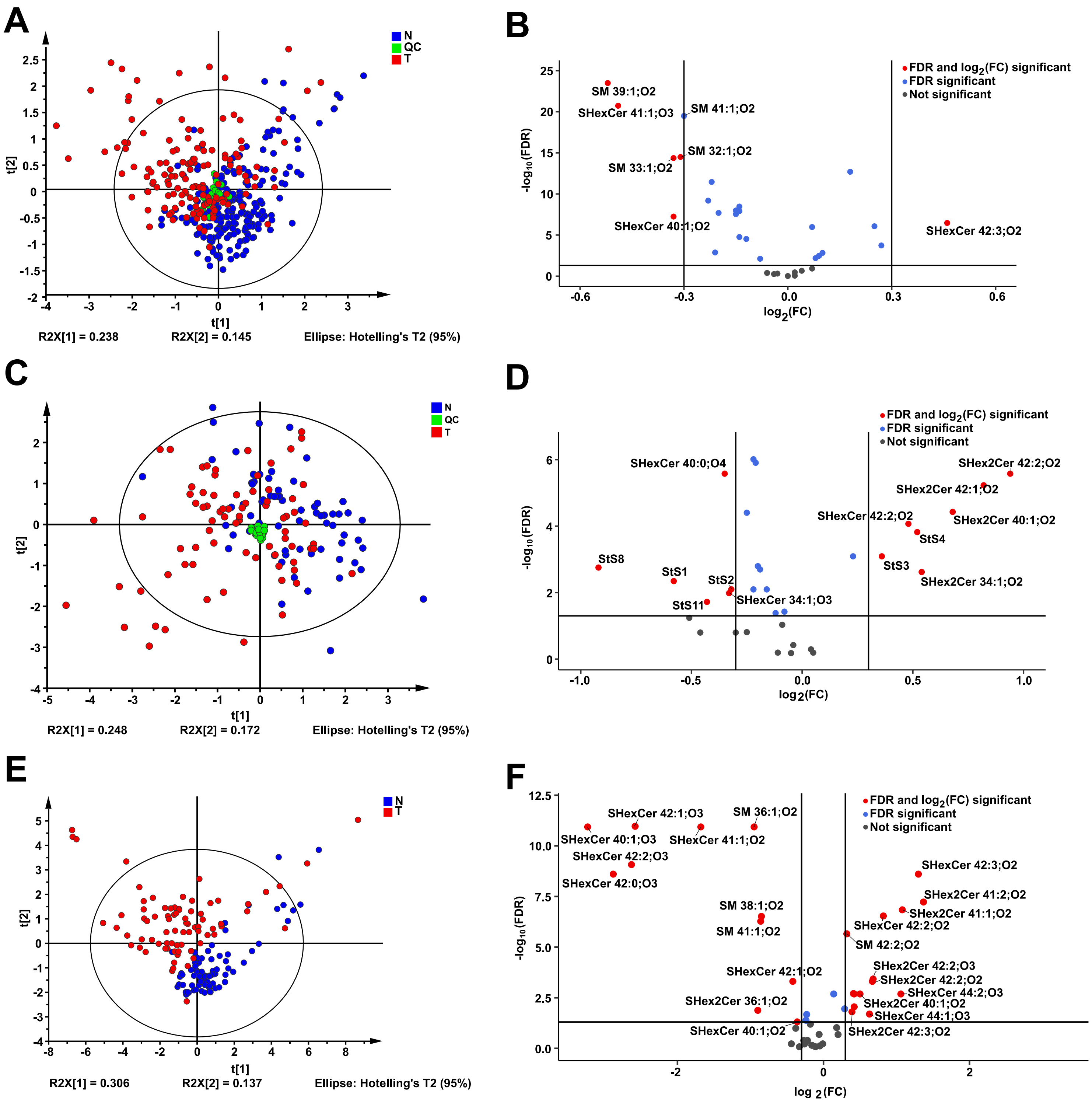

Figure S1. PCA-X score plots and volcano plots built based on the evaluation of relative concentrations of lipids in studied samples: (**A**) plasma (207 controls - blue, 143 cancer patients - red, and 18 QC - green), (**C**) urine (67 controls - blue, 76 cancer patients - red, and 18 QC - green), and (**E**) tissue (nonneoplastic (N) and tumor (T) tissues of 77 patients). Data were log-transformed and Pareto-scaled before the PCA analysis. Volcano plot reflects statistically most significant lipid species in studies (**B**) plasma, (**D**) urine, and (**F**) tissue samples with respect to highest fold change (robust Hodges-Lehmann type fold change estimator) and false discovery rate (FDR) from the Mann-Whitney U test (plasma, urine) or the Wilcoxon signed-rank test for paired samples (tissues).

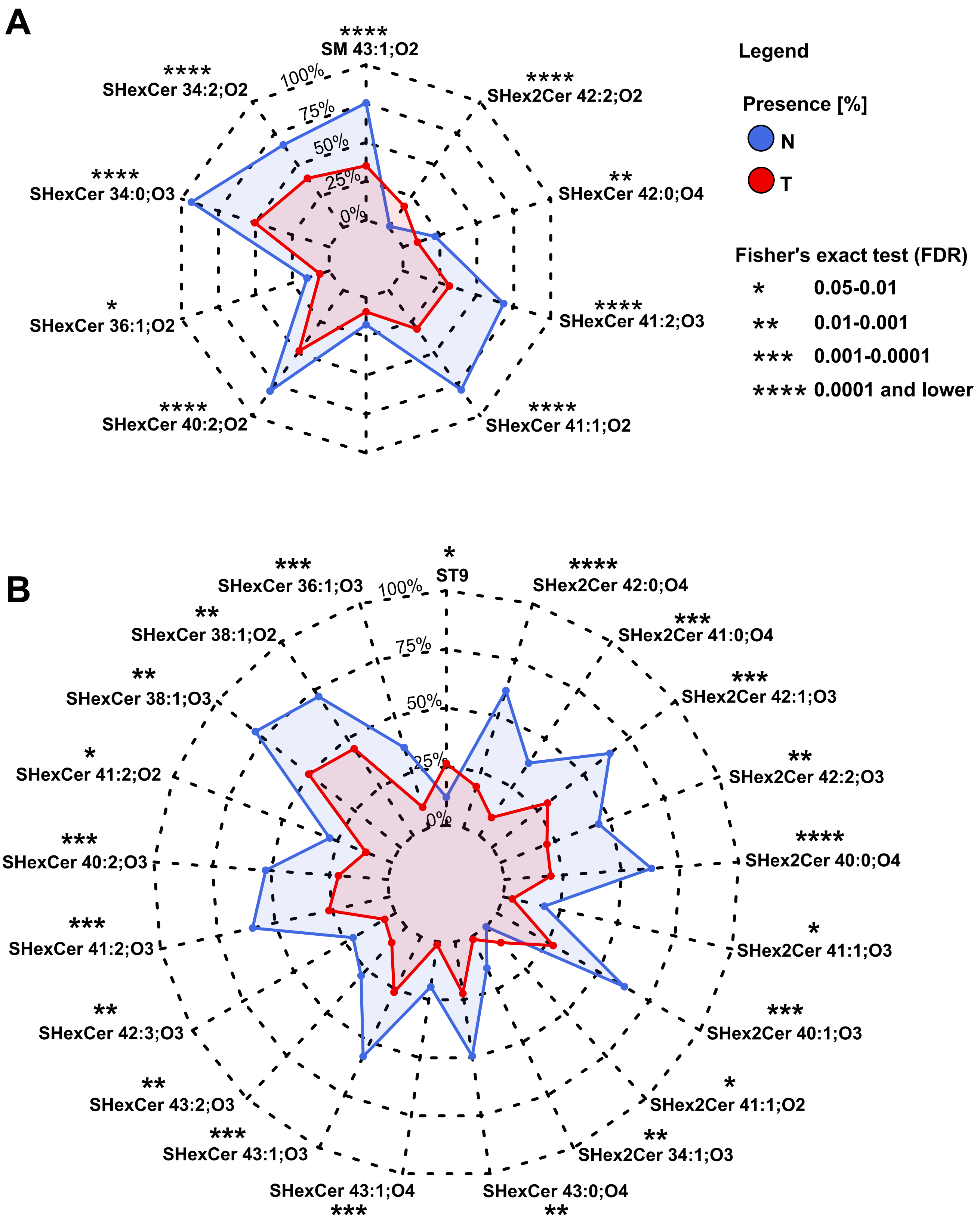

Figure S2. Radar charts of SHexCer, SHex2Cer, SM, and StS species analyzed using the present/absent approach for (**A**) plasma and (**B**) urine samples of controls (blue) and RCC patients (red). Contingency tables (Supplementary Data 2C, E) were created for these lipids, and Fisher's exact test was applied for the presence/absence analysis.

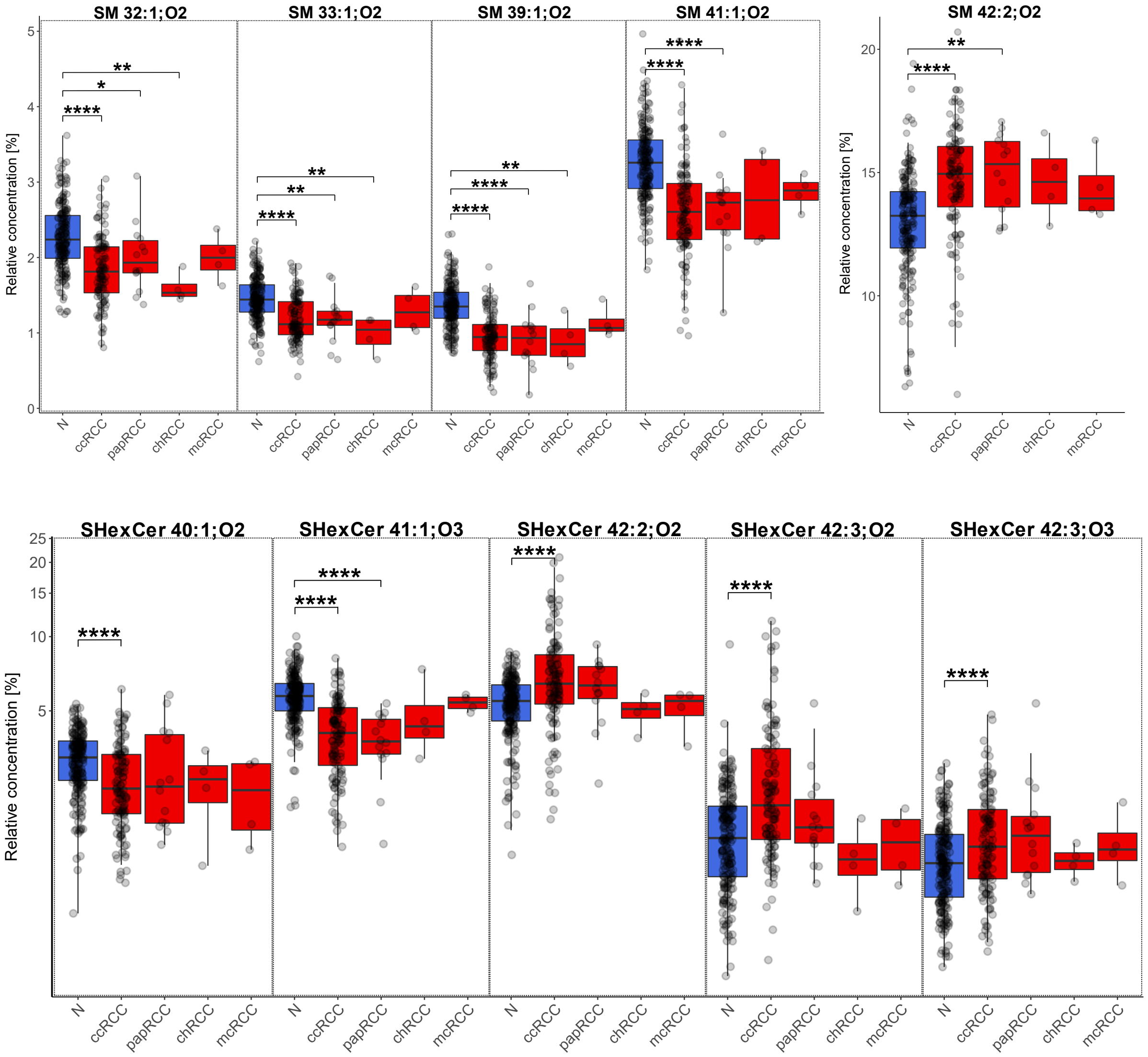

Supplementary Figure S3. Box plots illustrate differences between healthy controls and patients with different RCC subtypes in relative concentrations of selected sphingomyelins and sulfatides in plasma samples.

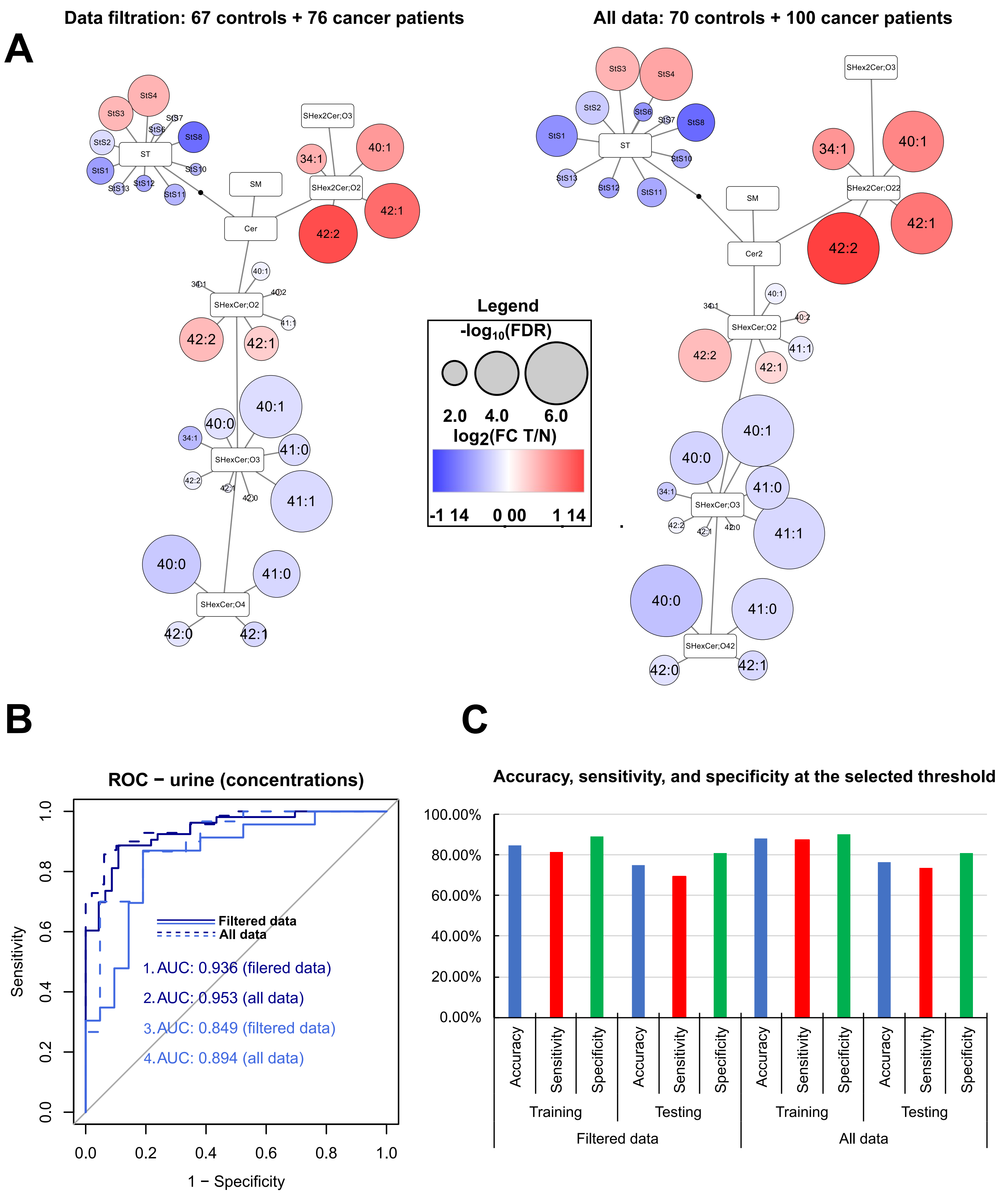

Supplementary Figure S4. (A) Cytoscape networks illustrate differences between the results of the Mann-Whitney U test and fold change analysis in two data sets obtained for urine lipidome profiling: for the data set with 27 subjects excluded and for the whole urine data set without any exclusions. The samples were excluded from further analysis based on the analytical discrepancies. (B) The ROC curves illustrate the performance of logistic regression in classifying RCC patients and healthy controls. The training and testing sets were obtained in the effect of splitting two data matrices: the first one - 27 subjects excluded (solid lines) and the second one - all subjects included (dashed lines). (C) The bar chart compares logistic regression's accuracy, sensitivity, and specificity for both data sets – on the left for the matrix with 27 subjects excluded, on the right for all subjects included.

A

Abbreviation	Theoretical m/z of $[M\text{-}H]^-$	Elemental composition	Suggested compound
StS 1	411.1847	$C_{21}H_{32}O_6S$	tetrahydrocorticosterone sulfate-H_2O
StS 2	429.1953	$C_{21}H_{34}O_7S$	tetrahydrocorticosterone sulfate
StS 3	441.1589	$C_{21}H_{30}O8S$	cortisol sulfate
StS 4	445.1902	$C_{21}H_{34}O_8S$	tetrahydrocortisol sulfate
StS 5	455.2473	$C_{24}H_{40}O_6S$	lithocholic acid sulfate
StS 6	465.304	$C_{27}H_{46}O_4S$	cholesterol sulfate
StS 7	471.2422	$C_{24}H_{40}O_7S$	ursodeoxycholic acid sulfate
StS 8	482.2946	$C_{26}H_{45}NO_5S$	taurolithocholic acid
StS 9	487.2371	$C_{24}H_{40}O_8S$	sulfocholic acid
StS 10	498.2895	$C_{26}H_{45}NO_6S$	taurodeoxycholic acid
StS 11	512.2688	$C_{26}H_{43}NO_7S$	sulfoglycolithocholic acid
StS 12	514.2844	$C_{26}H_{45}NO_7S$	taurocholic acid
StS 13	528.2637	$C_{26}H_{43}NO_8S$	glycochenodeoxycholic acid sulfate

B

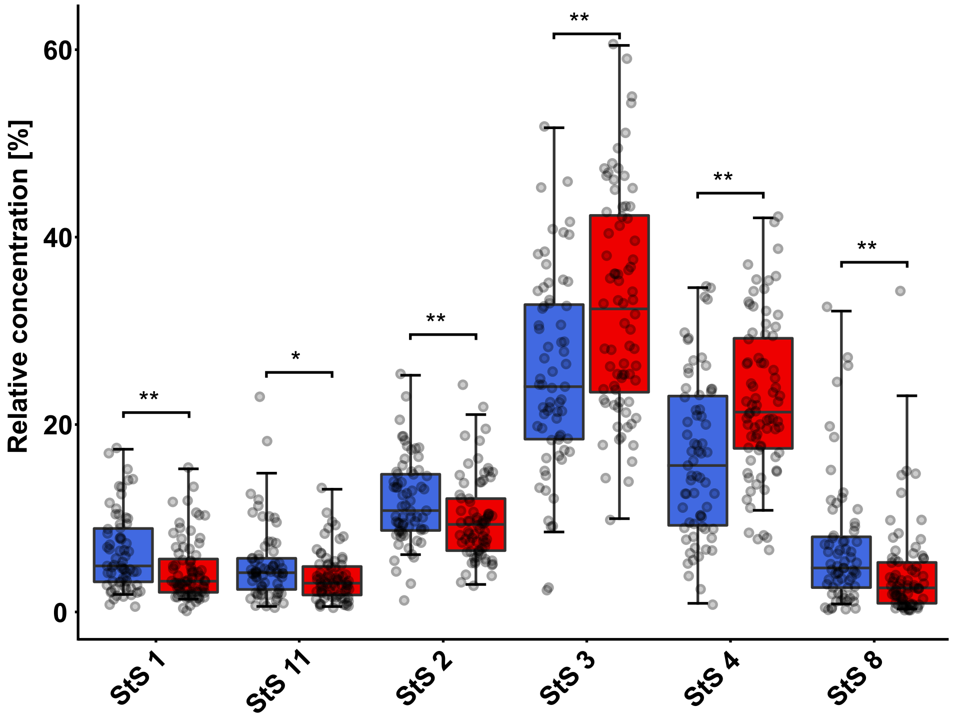

Figure S5. (**A**) Abbreviation of the sterols observed in urine samples with theoretical m/z value, elemental composition, and suggestion of the most probable corresponding compounds for individual elemental compositions. (**B**) Box plots (relative concentrations) of selected sterols measured in urine samples for controls (blue) and patients with RCC (red). The importance marked above box plots includes fold change, effect size, and FDR from the Mann-Whitney U test: **** - very large, *** - large, ** - medium, * - small significance (more explanation in the Supplementary Data 2D).

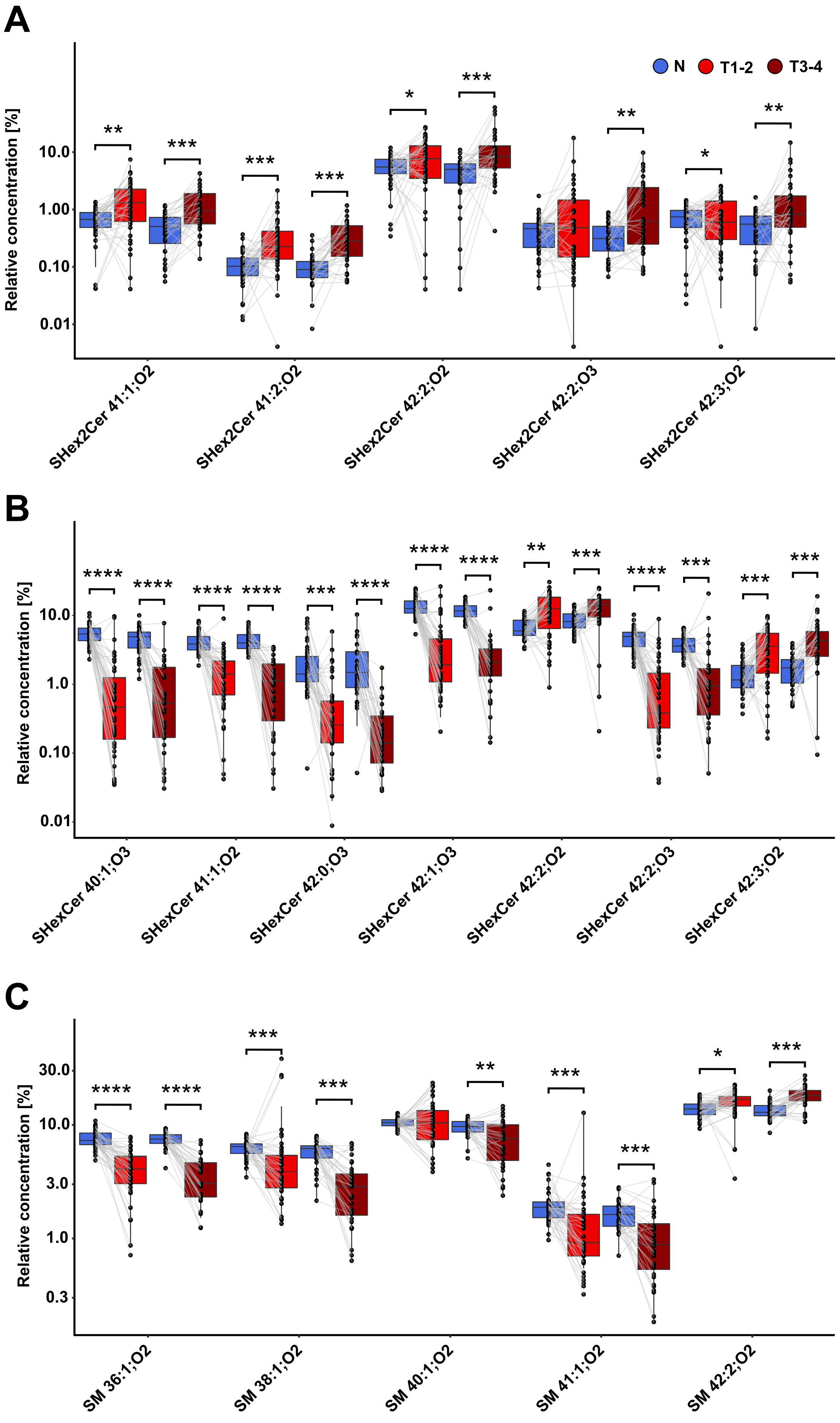

Figure S6. Comparison of the most statistically significant lipids in tumor tissue and surrounding nonneoplastic tissue of RCC patients with early tumor stage (T1-T2) and RCC patients with more advanced tumor stage (T3-T4). The importance marked above box plots includes fold change, effect size, and FDR from the Wilcoxon signed-rank test: **** - very large, *** - large, ** - medium, * - small significance (more explanation in the Supplementary Data 2L, M).

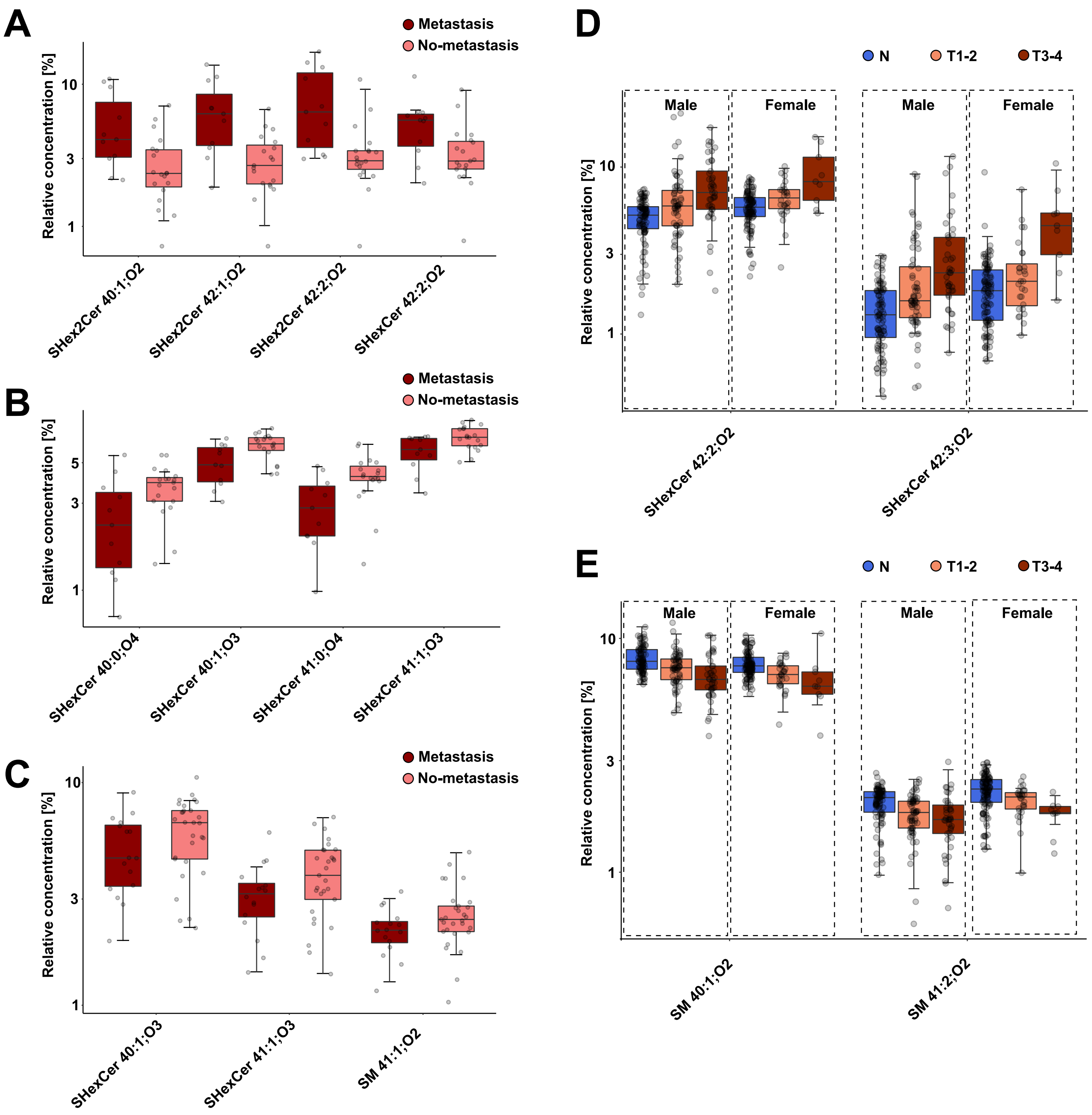

Figure S7. (**A**) Box plots present differences in relative concentrations of selected lipids between RCC patients with T3 tumor stage with (dark red) or without metastases (pink), measured in urine (**A**, **B**) and plasma (**C**). Box plots present subtle differences in the relative concentration of selected (**D**) SHexCer and (**E**) SM between genders in studied plasma samples (N = controls, T1-2 = patients with early tumor stage, T3-4 = patients with more advanced tumor stage).
